# Supplementary material for: Maternal dietary patterns, breastfeeding duration, and their association with child cognitive function and head circumference growth: A prospective mother–child cohort study
Source: PLoS Med. 2025 Apr 10;22(4):e1004454. doi: 10.1371/journal.pmed.1004454 (PMC11984734; doi:10.1371/journal.pmed.1004454)
Supplement: S1 Table — (DOCX) [file pmed.1004454.s001.docx]

| **FFQ nutrient constituents** | **Mean (SD)** | **PC1 loadings**  **“Varied Dietary Pattern”** | **PC2 loadings**  **“Western dietary Pattern”** |
| --- | --- | --- | --- |
| Niacin equivalents (NE/day) (mean (SD)) | 26.4 (6.4) | 0.142 | -0.045 |
| Ashes (g/day) (mean (SD)) | 20.2 (5.0) | 0.142 | -0.018 |
| Zink (mg/day) (mean (SD)) | 12.0 (3.0) | 0.141 | -0.03 |
| Alanine (mg/day) (mean (SD)) | 3405 (833) | 0.141 | -0.023 |
| Asparagine acid (mg/day) (mean (SD)) | 6347 (1583) | 0.141 | -0.021 |
| Histidine (mg/day) (mean (SD)) | 1924 (503) | 0.139 | 0.008 |
| Serine (mg/day) (mean (SD)) | 3421 (926) | 0.139 | 0.007 |
| Threonine (mg/day) (mean (SD)) | 2615 (699) | 0.139 | -0.002 |
| Arginine (mg/day) (mean (SD)) | 3815 (955) | 0.138 | -0.049 |
| Valine (mg/day) (mean (SD)) | 4098 (1106) | 0.138 | 0.005 |
| Tryptophan (mg/day) (mean (SD)) | 14.7 (4.0) | 0.138 | -0.004 |
| Phenylalanine (mg/day) (mean (SD)) | 3060 (833) | 0.138 | 0.004 |
| Tryptophan (mg/day) (mean (SD)) | 802 (217) | 0.138 | 0.004 |
| Selenium (µg/day) (mean (SD)) | 47.2 (12.6) | 0.137 | -0.052 |
| Glutamic acid (mg/day) (mean (SD)) | 12803 (3446) | 0.137 | 0.015 |
| Isoleucine (mg/day) (mean (SD)) | 3257 (895) | 0.137 | 0.009 |
| Glycine (mg/day) (mean (SD)) | 2919 (713) | 0.136 | -0.023 |
| Leucine (mg/day) (mean (SD)) | 5356 (1490) | 0.136 | 0.011 |
| Methionine (mg/day) (mean (SD)) | 1553 (422) | 0.136 | 0.007 |
| Vitamin B6 (mg/day) (mean (SD)) | 1.6 (0.4) | 0.135 | -0.111 |
| Phosphor (mg/day) (mean (SD)) | 1654 (461) | 0.135 | -0.03 |
| Pantothenic acid (mg/day) (mean (SD)) | 6.2 (1.7) | 0.134 | -0.049 |
| Cystine (mg/day) (mean (SD)) | 667 (184) | 0.134 | -0.028 |
| Tyrosine (mg/day) (mean (SD)) | 2296 (660) | 0.133 | 0.014 |
| Potassium (mg/day) (mean (SD)) | 3584 (942) | 0.131 | -0.083 |
| Lysine (mg/day) (mean (SD)) | 4772 (1353) | 0.131 | 0.006 |
| Vitamin B1 (thiamin) (mg/day) (mean (SD)) | 1.4 (0.4) | 0.13 | -0.063 |
| Proline (mg/day) (mean (SD)) | 4841 (1438) | 0.13 | 0.031 |
| Biotin (µg/day) (mean (SD)) | 42.9 (13.2) | 0.128 | -0.068 |
| Magnesium (mg/day) (mean (SD)) | 399 (108) | 0.126 | -0.106 |
| Niacin (mg/day) (mean (SD)) | 14.5 (3.7) | 0.126 | -0.074 |
| Iron (mg/day) (mean (SD)) | 11.2 (2.9) | 0.124 | -0.074 |
| Sodium (mg/day) (mean (SD)) | 2957 (813) | 0.124 | 0.043 |
| Vitamin B12 (µg/day) (mean (SD)) | 5.6 (2.1) | 0.116 | -0.017 |
| Folate (µg/day) (mean (SD)) | 345 (101) | 0.115 | -0.108 |
| Vitamin B2 (riboflavin) (mg/day) (mean (SD)) | 1.9 (0.7) | 0.115 | 0.002 |
| C18:1, n-9 (Oleic acid, Elaidic acid) (g/day) (mean (SD)) | 22.8 (7.6) | 0.112 | 0.125 |
| C16:1, n-7 (Palmitoleic acid) (g/day) (mean (SD)) | 1.3 (0.4) | 0.11 | 0.144 |
| C18:2, n-6 (Linoleic acid) (g/day) (mean (SD)) | 9.4 (3.2) | 0.108 | -0.019 |
| Chromium (µg/day) (mean (SD)) | 32.4 (10.3) | 0.107 | -0.058 |
| C18:3, n-3 (Alpha-linolenic acid ALA) (g/day) (mean (SD)) | 1.9 (0.7) | 0.107 | 0.018 |
| C16:0 (Palmitic acid, Hexadecanoic acid) (g/day) (mean (SD)) | 15.6 (5.3) | 0.106 | 0.202 |
| C20:1, n-11 (Gadoleic acid) (g/day) (mean (SD)) | 0.52 (0.21) | 0.105 | 0.113 |
| Cholesterol (mg/day) (mean (SD)) | 307 (111) | 0.104 | 0.143 |
| Calcium (mg/day) (mean (SD)) | 1238 (454) | 0.103 | 0.005 |
| Vitamin E (a-TE/day) (mean (SD)) | 7.9 (3.0) | 0.099 | -0.045 |
| Dietary fiber (g/day) (mean (SD)) | 27.9 (9.8) | 0.097 | -0.125 |
| Alphatocoferol (mg/day) (mean (SD)) | 7.3 (2.8) | 0.097 | -0.044 |
| C18:0 (Stearic acid, Octadecanoic acid) (g/day) (mean (SD)) | 6.7 (2.6) | 0.095 | 0.195 |
| C12:0 (Lauric acid, Dodecanoic acid) (g/day) (mean (SD)) | 1.3 (0.5) | 0.093 | 0.215 |
| Iodine (µg/day) (mean (SD)) | 302 (120) | 0.092 | -0.02 |
| C14:0 (Myristic acid, Tetradecanoic acid) (g/day) (mean (SD)) | 3.2 (1.4) | 0.09 | 0.233 |
| Manganese (mg/day) (mean (SD)) | 5.2 (2.1) | 0.09 | -0.122 |
| Starch (g/day) (mean (SD)) | 100 (35) | 0.09 | -0.022 |
| C18:1, n-7 (Vaccenic acid) (g/day) (mean (SD)) | 0.12 (0.05) | 0.089 | -0.049 |
| Nickel (µg/day) (mean (SD)) | 150 (53) | 0.088 | -0.115 |
| Other fatty acids (g/day) (mean (SD)) | 0.17 (0.07) | 0.087 | -0.073 |
| Retinol (µg/day) (mean (SD)) | 491 (241) | 0.085 | 0.134 |
| Water (g/day) (mean (SD)) | 2817 (740) | 0.085 | -0.072 |
| Vitamin A (RE/day) (mean (SD)) | 825 (369) | 0.085 | 0.01 |
| Trans fatty acids, total (g/day) (mean (SD)) | 1.6 (0.8) | 0.084 | 0.24 |
| C14:1, n-5 (Myristoleic acid) (g/day) (mean (SD)) | 0.37 (0.17) | 0.083 | 0.231 |
| C10:0 (Capric acid, Decanoic acid) (g/day) (mean (SD)) | 0.84 (0.46) | 0.082 | 0.243 |
| C4:0 (Butyric acid, Butanoic acid) (g/day) (mean (SD)) | 0.97 (0.54) | 0.081 | 0.244 |
| C20:0 (Arachidic acid, Eicosanoic acid) (g/day) (mean (SD)) | 0.11 (0.06) | 0.08 | 0.154 |
| Vitamin D3 (cholecalciferol) (µg/day) (mean (SD)) | 1.1 (0.7) | 0.08 | -0.03 |
| C6:0 (Caproic acid, Hexanoic acid) (g/day) (mean (SD)) | 1.0 (0.6) | 0.079 | 0.243 |
| C8:0 (Caprylic acid, Octanoic acid) (g/day) (mean (SD)) | 0.57 (0.33) | 0.079 | 0.232 |
| C20:4, n-6 (Arachidonic acid AA) (g/day) (mean (SD)) | 0.05 (0.02) | 0.074 | -0.026 |
| Lactose (g/day) (mean (SD)) | 23.3 (15.9) | 0.073 | 0.028 |
| Vitamin D (µg/day) (mean (SD)) | 4.9 (2.8) | 0.071 | -0.087 |
| C22:6, n-3 (Docosahexaenoic acid DHA, Cervonic acid) (g/day) (mean (SD)) | 0.36 (0.24) | 0.069 | -0.102 |
| C20:5, n-3 (Eicosapentaenoic acid EPA, Timnodonic acid) (g/day) (mean (SD)) | 0.15 (0.11) | 0.064 | -0.117 |
| L-ascorbic acid (mg/day) (mean (SD)) | 136 (66) | 0.063 | -0.14 |
| C22:5, n-3 (Docosapentaenoic acid DPA, Clupanodonic acid) (g/day) (mean (SD)) | 0.04 (0.03) | 0.063 | -0.097 |
| Vitamin C (mg/day) (mean (SD)) | 140 (71) | 0.061 | -0.138 |
| C24:1, n-9 (Nervonic acid) (g/day) (mean (SD)) | 0.02 (0.02) | 0.061 | -0.09 |
| C18:4, n-3 (Stearidonic acid SDA, Moroctic acid) (g/day) (mean (SD)) | 0.05 (0.05) | 0.057 | -0.08 |
| Sucrose (g/day) (mean (SD)) | 28.0 (12.1) | 0.056 | -0.01 |
| Maltose (g/day) (mean (SD)) | 2.3 (1.2) | 0.056 | 0.01 |
| Copper (mg/day) (mean (SD)) | 4.9 (1.5) | 0.055 | -0.095 |
| Glucose (g/day) (mean (SD)) | 13.0 (6.5) | 0.052 | -0.135 |
| Vitamin K (µg/day) (mean (SD)) | 86.5 (53.0) | 0.052 | -0.102 |
| Fructose (g/day) (mean (SD)) | 13.7 (6.8) | 0.05 | -0.158 |
| L-dehydroascorbic acid (mg/day) (mean (SD)) | 13.8 (7.4) | 0.05 | -0.115 |
| C22:1, n-11 (Cetoleic acid) (g/day) (mean (SD)) | 0.14 (0.17) | 0.05 | -0.063 |
| 25-hydroxycholecalciferol - a metabolically activated form of cholecalciferol (Vitamin D3) (µg/day) (mean (SD)) | 0.03 (0.02) | 0.048 | 0.086 |
| C22:0 (Behenic acid, Docosanoic acid) (g/day) (mean (SD)) | 0.05 (0.04) | 0.046 | 0.01 |
| C17:0 (Margaric acid, Heptadecanoic acid) (g/day) (mean (SD)) | 0.02 (0.01) | 0.043 | -0.006 |
| C15:0 (Pentadecylic acid, Pentadecanoic acid) (g/day) (mean (SD)) | 0.01 (0.01) | 0.041 | -0.018 |
| C24:0 (Lignoceric acid, Tetracosanoic acid) (g/day) (mean (SD)) | 0.03 (0.03) | 0.039 | -0.091 |
| Betacarotene (µg/day) (mean (SD)) | 3958 (3258) | 0.038 | -0.106 |
| Added sugar (g/day) (mean (SD)) | 36.0 (26.2) | 0.033 | 0.123 |
| C22:1, n-9 (Erucic acid) (g/day) (mean (SD)) | 0.02 (0.04) | 0.028 | -0.041 |
| Alcohol (g/day) (mean (SD)) | 0.41 (0.75) | 0.015 | 0.013 |

**S1 Table: Nutrient Constituents and Principal Component Loadings for Dietary Patterns.** This table presents the nutrient constituents used in the principal component analysis (PCA) to define the "Varied" and "Western" dietary patterns during pregnancy. It includes the mean and standard deviation (SD) of nutrient intakes in the population, along with their corresponding principal component loadings for PC1 (Varied dietary pattern) and PC2 (Western dietary pattern). The table provides a comprehensive overview of the nutrient contributions to each dietary pattern, highlighting the key nutrients associated with each pattern. This information supports the interpretation of the dietary patterns and their associations with FFQ-derived food groups.
