# Supplementary material for: Maternal dietary patterns, breastfeeding duration, and their association with child cognitive function and head circumference growth: A prospective mother–child cohort study
Source: PLoS Med. 2025 Apr 10;22(4):e1004454. doi: 10.1371/journal.pmed.1004454 (PMC11984734; doi:10.1371/journal.pmed.1004454)
Supplement: S2 Table — (DOCX) [file pmed.1004454.s002.docx]

| **Cohort Characteristics** | **Participated** | **Not Participated** | **p-value** |
| --- | --- | --- | --- |
| n = | 600 | 95 |  |
| Age (mean (SD)) | 10.3 (0.4) | - | - |
| Male Sex (%) | 310 (51.7) | 47 (49.5) | 0.774 |
| Caucasian Race (%) | 573 (95.5) | 93 (97.9) | 0.419 |
| Income type (%) |  |  | 0.159 |
| Low (<50,000 euro) | 48 ( 8.0) | 12 (12.6) |  |
| Medium (50,000 - 110,000 euro) | 320 (53.3) | 54 (56.8) |  |
| High (>110,000 euro) | 232 (38.7) | 29 (30.5) |  |
| Maternal Education Level at Birth |  |  | 0.205 |
| Low (primary, secondary, or college graduate) | 46 ( 7.7) | 5 ( 5.3) |  |
| Medium (tradesman or bachelor's degree) | 373 (62.2) | 68 (71.6) |  |
| High (Masters degree) | 181 (30.2) | 22 (23.2) |  |
| Maternal age at birth (mean (SD)) | 32.3 (4.3) | 31.8 (4.5) | 0.278 |
| Birthweight (mean (SD)) | 3.54 (0.54) | 3.53 (0.56) | 0.766 |
| Gestational age (mean (SD)) | 279 (12) | 278 (11) | 0.417 |
| Caesarean section (%) | 125 (20.8) | 25 (26.3) | 0.283 |
| Maternal smoking during pregnancy (%) | 44 ( 7.3) | 10 (10.5) | 0.382 |
| Solely Breastfed (mean (SD)) | 105 (59) | 94 (65) | 0.122 |
| Breastfeeding duration (mean (SD))) | 249 (162) | 235 (188) | 0.457 |
| Siblings (mean (SD)) | 1.5 (0.9) | 1.4 (0.9) | 0.578 |

**S2 Table**: **Baseline Characteristics and Cognition Scores Stratified by Participation in COPSYCH 10 Year Clinical Visit.** This table presents the baseline characteristic, stratified by those who participated and those who did not participate in the COPSYCH 10 year clinical visit. It provides a comprehensive overview of the cohort's characteristics, highlighting differences between participants and non-participants at the 10 year visit (86.3% follow-up).
