# Supplementary material for: Maternal dietary patterns, breastfeeding duration, and their association with child cognitive function and head circumference growth: A prospective mother–child cohort study
Source: PLoS Med. 2025 Apr 10;22(4):e1004454. doi: 10.1371/journal.pmed.1004454 (PMC11984734; doi:10.1371/journal.pmed.1004454)
Supplement: S3 Table — (DOCX) [file pmed.1004454.s003.docx]

| **Cohort Characteristics** | **Female** | **Male** | **p-value** |
| --- | --- | --- | --- |
| n = | 338 | 357 |  |
| Age (mean (SD)) | 10.3 (0.4) | 10.3 (0.4) | 0.233 |
| Caucasian Race (%) | 325 (96.2) | 341 (95.5) | 0.819 |
| Income type (%) |  |  | 0.718 |
| Low (<50,000 euro) | 32 (9.5) | 28 (7.8) |  |
| Medium (50,000 - 110,000 euro) | 182 (53.8) | 192 (53.8) |  |
| High (>110,000 euro) | 124 (36.7) | 137 (38.4) |  |
| Maternal Education Level at Birth |  |  | 0.256 |
| Low (primary, secondary, or college graduate) | 27 (8.0) | 24 (6.7) |  |
| Medium (tradesman or bachelor's degree) | 204 (60.4) | 237 (66.4) |  |
| High (Masters degree) | 107 (31.7) | 96 (26.9) |  |
| Maternal age at birth (mean (SD)) | 32.3 (4.2) | 32.2 (4.5) | 0.703 |
| Birthweight (mean (SD)) | 3.50 (0.53) | 3.58 (0.56) | 0.06 |
| Gestational age (mean (SD)) | 280 (11) | 279 (12) | 0.178 |
| Caesarean section (%) | 67 (19.8) | 83 (23.2) | 0.315 |
| Maternal smoking during pregnancy (%) | 26 (7.7) | 28 (7.8) | 1 |
| Solely Breastfed (mean (SD)) | 104 (60) | 103 (59) | 0.881 |
| Breastfeeding duration (mean (SD))) | 255 (153) | 240 (177) | 0.242 |
| Siblings (mean (SD)) | 1.4 (0.9) | 1.5 (0.9) | 0.473 |
| Bayley-III Cognitive Composite score (mean (SD)) | 105.9 (10.3) | 103.7 (9.1) | 0.004 |
| WISC-IV Full Scale Intelligence Quotient (mean (SD)) | 104.5 (11.3) | 101.1 (12.6) | 0.001 |

**S3 Table. Baseline Characteristics and Cognition Scores Stratified by Child Sex.** This table presents the baseline characteristics and cognition scores, stratified by child sex (females n=338 and males n=357). It provides a comprehensive overview of the cohort's characteristics, highlighting differences between females and males.
