# Supplementary material for: Maternal dietary patterns, breastfeeding duration, and their association with child cognitive function and head circumference growth: A prospective mother–child cohort study
Source: PLoS Med. 2025 Apr 10;22(4):e1004454. doi: 10.1371/journal.pmed.1004454 (PMC11984734; doi:10.1371/journal.pmed.1004454)
Supplement: S4 Table — (DOCX) [file pmed.1004454.s004.docx]

| **Cognitive Scores** | **Univariate Model** | **Multivariable Model** | **Multivariable with PRS** |
| --- | --- | --- | --- |
| **Western Dietary Pattern Metabolite Score** | **Estimate [95% Cl] p-value** | **Estimate [95% Cl] p-value** | **Estimate [95% Cl] p-value** |
| WISC: General Ability Index | -2.7 [-3.85, -1.56] (p < 0.001) | -1.02 [-2.33, 0.3] (p = 0.129) | -1.03 [-2.31, 0.25] (p = 0.116) |
| WISC: Verbal comprehension Index | -2.91 [-4.01, -1.81] (p < 0.001) | -1.48 [-2.77, -0.2] (p = 0.024) | -1.5 [-2.76, -0.24] (p = 0.02) |
| WISC: Perceptual reasoning Index | -1.81 [-3.16, -0.45] (p = 0.009) | -0.17 [-1.72, 1.39] (p = 0.834) | -0.18 [-1.72, 1.36] (p = 0.819) |
| WISC: Processing speed Index | -0.61 [-1.56, 0.34] (p = 0.208) | -0.29 [-1.41, 0.82] (p = 0.607) | -0.32 [-1.43, 0.8] (p = 0.58) |
| WISC: Working memory Index | -2.06 [-3, -1.12] (p < 0.001) | -0.9 [-2, 0.19] (p = 0.106) | -0.9 [-1.99, 0.18] (p = 0.104) |
| **Varied Dietary Pattern Metabolite Score** | **Estimate [95% Cl] p-value** | **Estimate [95% Cl] p-value** | **Estimate [95% Cl] p-value** |
| WISC: General Ability Index | 2.51 [1.34, 3.67] (p < 0.001) | 1.27 [0.06, 2.48] (p = 0.04) | 1.12 [-0.07, 2.3] (p = 0.065) |
| WISC: Verbal comprehension Index | 2.9 [1.79, 4.01] (p < 0.001) | 1.92 [0.75, 3.1] (p = 0.001) | 1.8 [0.64, 2.96] (p = 0.002) |
| WISC: Perceptual reasoning Index | 1.45 [0.08, 2.81] (p = 0.038) | 0.19 [-1.24, 1.62] (p = 0.796) | 0.05 [-1.37, 1.46] (p = 0.946) |
| WISC: Processing speed Index | 0.8 [-0.15, 1.76] (p = 0.101) | 0.58 [-0.44, 1.61] (p = 0.267) | 0.57 [-0.46, 1.59] (p = 0.279) |
| WISC: Working memory Index | 2.11 [1.16, 3.05] (p < 0.001) | 1.23 [0.23, 2.24] (p = 0.016) | 1.15 [0.15, 2.15] (p = 0.024) |
| **Duration of Breastfeeding *** | **Estimate [95% Cl] p-value** | **Estimate [95% Cl] p-value** | **Estimate [95% Cl] p-value** |
| WISC: General Ability Index | 1.72 [0.52, 2.93] (p = 0.005) | 0.62 [-0.59, 1.82] (p = 0.318) | 0.78 [-0.4 , 1.96] (p = 0.194) |
| WISC: Verbal comprehension Index | 2.05 [0.9, 3.2] (p = 0.001) | 1.15 [-0.02, 2.32] (p = 0.054) | 1.31 [0.16, 2.45] (p = 0.026) |
| WISC: Perceptual reasoning Index | 1.01 [-0.4, 2.42] (p = 0.161) | -0.07 [-1.5, 1.37] (p = 0.929) | 0.07 [-1.35, 1.49] (p = 0.922) |
| WISC: Processing speed Index | 0.33 [-0.65, 1.31] (p = 0.505) | -0.01 [-1.02, 1.01] (p = 0.986) | 0.01 [-1, 1.03] (p = 0.982) |
| WISC: Working memory Index | 1.1 [0.11, 2.09] (p = 0.03) | 0.23 [-0.78, 1.24] (p = 0.657) | 0.3 [-0.71, 1.3] (p = 0.56) |

**S4 Table. Associations between Western Dietary Pattern Metabolite Score and WISC-IV Composite Scores.** This table presents the results of linear regression analyses assessing the associations between a Western dietary pattern metabolite score during pregnancy and WISC-IV cognitive outcomes at 10 years. Estimates are interpreted as the effect of a 1 standard deviation increase of a Western dietary pattern metabolite score. The table provides both unadjusted and adjusted associations, with the latter controlling for potential confounders such as pre-pregnancy maternal body mass index, child sex, birth weight, gestational age, smoking during pregnancy, antibiotic use during pregnancy, pre-eclampsia, household income at birth, maternal education level at birth, maternal age at birth and breastfeeding duration. Further adjustments include maternal and child polygenic risk score for intelligence, and child head circumference polygenic risk scores.

*** Note breastfeeding is log-transformed and z-scored, thus estimates are interpreted as per 1 SD change.**
