# Supplementary material for: Maternal dietary patterns, breastfeeding duration, and their association with child cognitive function and head circumference growth: A prospective mother–child cohort study
Source: PLoS Med. 2025 Apr 10;22(4):e1004454. doi: 10.1371/journal.pmed.1004454 (PMC11984734; doi:10.1371/journal.pmed.1004454)
Supplement: S5 Table — (DOCX) [file pmed.1004454.s005.docx]

| **Cognitive Score** | **Prenatal RCTs** | **Child Western Dietary Pattern** | **Neurodevelopmental Diagnosis** | **Parental Head Circumference** |
| --- | --- | --- | --- | --- |
| **Western Dietary Pattern Metabolite Score** | **Estimate [95% Cl] p-value** | **Estimate [95% Cl] p-value** | **Estimate [95% Cl] p-value** | **Estimate [95% Cl] p-value** |
| Bayley-III Composite Score | -1.15 [-2.08, -0.23] (p = 0.015) | -1.13 [-2.05, -0.21] (p = 0.017) | -1.18 [-2.1, -0.26] (p = 0.012) | -1.24 [-2.15, -0.33] (p = 0.008) |
| WISC: Full Scale Intelligence Quotient | -1.06 [-2.17, 0.06] (p = 0.063) | -0.9 [-2.02, 0.21] (p = 0.113) | -0.78 [-1.89, 0.32] (p = 0.166) | -0.98 [-2.09, 0.12] (p = 0.083) |
| WISC: General Ability Index | -1.22 [-2.55, 0.1] (p = 0.07) | -1.01 [-2.34, 0.31] (p = 0.135) | -0.9 [-2.22, 0.42] (p = 0.183) | -1.03 [-2.35, 0.29] (p = 0.126) |
| WISC: Verbal Comprehension Index | -1.79 [-3.07, -0.51] (p = 0.006) | -1.38 [-2.68, -0.09] (p = 0.037) | -1.34 [-2.63, -0.05] (p = 0.042) | -1.49 [-2.78, -0.2] (p = 0.024) |
| WISC: Perceptual reasoning Index | -0.22 [-1.79, 1.35] (p = 0.784) | -0.26 [-1.83, 1.31] (p = 0.743) | -0.12 [-1.69, 1.44] (p = 0.878) | -0.18 [-1.74, 1.38] (p = 0.82) |
| WISC: Processing speed Index | -0.09 [-1.21, 1.03] (p = 0.876) | -0.19 [-1.32, 0.93] (p = 0.738) | -0.09 [-1.21, 1.02] (p = 0.87) | -0.32 [-1.44, 0.79] (p = 0.569) |
| WISC: Working memory Index | -0.97 [-2.07, 0.14] (p = 0.086) | -0.83 [-1.94, 0.27] (p = 0.14) | -0.77 [-1.87, 0.32] (p = 0.167) | -0.94 [-2.03, 0.15] (p = 0.092) |
| **Varied Dietary Pattern Metabolite Score** | **Estimate [95% Cl] p-value** | **Estimate [95% Cl] p-value** | **Estimate [95% Cl] p-value** | **Estimate [95% Cl] p-value** |
| Bayley-III Composite Score | 0.02 [-0.81, 0.86] (p = 0.956) | -0.02 [-0.86, 0.81] (p = 0.956) | 0.02 [-0.82, 0.86] (p = 0.964) | 0.04 [-0.8, 0.88] (p = 0.926) |
| WISC: Full Scale Intelligence Quotient | 1.29 [0.27, 2.31] (p = 0.013) | 1.26 [0.25, 2.28] (p = 0.015) | 1.28 [0.28, 2.29] (p = 0.013) | 1.27 [0.25, 2.29] (p = 0.015) |
| WISC: General Ability Index | 1.25 [0.04, 2.45] (p = 0.043) | 1.26 [0.05, 2.47] (p = 0.041) | 1.27 [0.07, 2.48] (p = 0.039) | 1.26 [0.05, 2.47] (p = 0.042) |
| WISC: Verbal Comprehension Index | 1.89 [0.72, 3.06] (p = 0.002) | 1.89 [0.71, 3.07] (p = 0.002) | 1.93 [0.76, 3.1] (p = 0.001) | 1.92 [0.74, 3.1] (p = 0.001) |
| WISC: Perceptual reasoning Index | 0.18 [-1.25, 1.62] (p = 0.801) | 0.21 [-1.22, 1.65] (p = 0.77) | 0.19 [-1.24, 1.62] (p = 0.795) | 0.17 [-1.26, 1.61] (p = 0.812) |
| WISC: Processing speed Index | 0.65 [-0.37, 1.67] (p = 0.213) | 0.55 [-0.48, 1.57] (p = 0.294) | 0.58 [-0.44, 1.59] (p = 0.267) | 0.56 [-0.47, 1.58] (p = 0.285) |
| WISC: Working memory Index | 1.25 [0.25, 2.25] (p = 0.015) | 1.21 [0.21, 2.22] (p = 0.018) | 1.24 [0.24, 2.24] (p = 0.016) | 1.2 [0.2, 2.2] (p = 0.019) |
| **Duration of Breastfeeding *** | **Estimate [95% Cl] p-value** | **Estimate [95% Cl] p-value** | **Estimate [95% Cl] p-value** | **Estimate [95% Cl] p-value** |
| Bayley-III Composite Score | -0.22 [-1.07, 0.62] (p = 0.606) | -0.29 [-1.14, 0.56] (p = 0.507) | -0.23 [-1.08, 0.62] (p = 0.592) | -0.25 [-1.09, 0.6] (p = 0.566) |
| WISC: Full Scale Intelligence Quotient | 0.37 [-0.65, 1.39] (p = 0.476) | 0.39 [-0.63, 1.41] (p = 0.454) | 0.46 [-0.55, 1.47] (p = 0.371) | 0.43 [-0.59, 1.44] (p = 0.413) |
| WISC: General Ability Index | 0.54 [-0.67, 1.75] (p = 0.38) | 0.6 [-0.61, 1.82] (p = 0.329) | 0.64 [-0.56, 1.85] (p = 0.296) | 0.62 [-0.59, 1.83] (p = 0.315) |
| WISC: Verbal Comprehension Index | 1.06 [-0.11, 2.22] (p = 0.076) | 1.09 [-0.08, 2.26] (p = 0.068) | 1.19 [0.03, 2.36] (p = 0.045) | 1.16 [-0.02, 2.33] (p = 0.054) |
| WISC: Perceptual reasoning Index | -0.09 [-1.54, 1.35] (p = 0.9) | -0.03 [-1.47, 1.42] (p = 0.973) | -0.06 [-1.5, 1.38] (p = 0.935) | -0.06 [-1.5, 1.38] (p = 0.933) |
| WISC: Processing speed Index | 0.02 [-0.99, 1.04] (p = 0.964) | -0.06 [-1.08, 0.95] (p = 0.901) | 0.03 [-0.98, 1.04] (p = 0.951) | -0.01 [-1.02, 1.01] (p = 0.986) |
| WISC: Working memory Index | 0.17 [-0.84, 1.17] (p = 0.743) | 0.19 [-0.82, 1.21] (p = 0.71) | 0.26 [-0.75, 1.27] (p = 0.618) | 0.21 [-0.8, 1.22] (p = 0.689) |

**S5 Table: Subanalysis; Multivariable Regression Modelling of Dietary Exposures and Cognitive Outcomes.** This table presents the results of multivariable regression analyses assessing the associations between a Western and Varied dietary pattern metabolite score during pregnancy, breastfeeding duration and cognitive outcomes; specifically, the Bayleys-III Composite score at 2.5 years and WISC at 10 years. As a sensitivity analysis, we further added; n-3 LCPUFA and Vitamin D prenatal randomised control trials, a child Western dietary pattern as assessed via an independent 10 year food frequency questionnaire, parents head circumference measurements and any neurodevelopmental disorder diagnosis at age 10 years, as covariates. The estimates are interpreted as the effect of a 1 standard deviation change in dietary pattern metabolite scores and breastfeeding duration during pregnancy.

*** Note breastfeeding is log-transformed and z-scored, thus estimates are interpreted as per 1 SD change.**
