# Supplementary material for: Maternal dietary patterns, breastfeeding duration, and their association with child cognitive function and head circumference growth: A prospective mother–child cohort study
Source: PLoS Med. 2025 Apr 10;22(4):e1004454. doi: 10.1371/journal.pmed.1004454 (PMC11984734; doi:10.1371/journal.pmed.1004454)
Supplement: S6 Table — (DOCX) [file pmed.1004454.s006.docx]

| **Cognitive Scores** | **Female** | **Male** | **Interaction** |
| --- | --- | --- | --- |
| **Western Dietary Pattern Metabolite Score** | **Estimate [95% Cl] p-value** | **Estimate [95% Cl] p-value** | **Estimate [95% Cl] p-value** |
| Bayley-III Composite Score | -1.4 [-2.83, 0.03] (p = 0.056) | -1.21 [-2.38, -0.04] (p = 0.043) | 0.65 [-0.86, 2.15] (p = 0.401) |
| WISC: Full Scale Intelligence Quotient | -0.42 [-1.86, 1.02] (p = 0.567) | -1.75 [-3.45, -0.05] (p = 0.044) | -0.79 [-2.65, 1.08] (p = 0.407) |
| WISC: General Index | -0.59 [-2.37, 1.18] (p = 0.515) | -1.75 [-3.72, 0.22] (p = 0.083) | -0.12 [-2.33, 2.1] (p = 0.917) |
| WISC: Verbal comprehension Index | -0.79 [-2.53, 0.95] (p = 0.373) | -2.41 [-4.34, -0.47] (p = 0.015) | -0.85 [-3.01, 1.32] (p = 0.444) |
| WISC: Perceptual reasoning Index | -0.18 [-2.25, 1.89] (p = 0.866) | -0.45 [-2.78, 1.87] (p = 0.703) | 0.7 [-1.92, 3.33] (p = 0.599) |
| WISC: Processing speed Index | 0.5 [-1.06, 2.07] (p = 0.53) | -1.16 [-2.74, 0.42] (p = 0.153) | -2.14 [-4.01, -0.27] (p = 0.025) |
| WISC: Working memory Index | -0.77 [-2.18, 0.64] (p = 0.287) | -1.19 [-2.87, 0.49] (p = 0.166) | -0.5 [-2.34, 1.34] (p = 0.595) |
| **Varied Dietary Pattern Metabolite Score** | **Estimate [95% Cl] p-value** | **Estimate [95% Cl] p-value** | **Estimate [95% Cl] p-value** |
| Bayley-III Composite Score | -0.19 [-1.48, 1.1] (p = 0.776) | 0.24 [-0.85, 1.33] (p = 0.668) | 0.22 [-1.33, 1.76] (p = 0.784) |
| WISC: Full Scale Intelligence Quotient | 0.83 [-0.49, 2.15] (p = 0.22) | 2 [0.43, 3.58] (p = 0.013) | 1.23 [-0.66, 3.12] (p = 0.203) |
| WISC: General Index | 0.77 [-0.85, 2.4] (p = 0.351) | 1.95 [0.12, 3.78] (p = 0.037) | 0.76 [-1.49, 3.01] (p = 0.507) |
| WISC: Verbal comprehension Index | 1.43 [-0.15, 3.01] (p = 0.078) | 2.76 [0.97, 4.55] (p = 0.003) | 1.22 [-0.96, 3.41] (p = 0.274) |
| WISC: Perceptual reasoning Index | -0.12 [-2.01, 1.77] (p = 0.899) | 0.51 [-1.65, 2.67] (p = 0.643) | 0.05 [-2.62, 2.71] (p = 0.972) |
| WISC: Processing speed Index | -0.01 [-1.44, 1.43] (p = 0.992) | 1.44 [-0.02, 2.91] (p = 0.055) | 2.25 [0.35, 4.15] (p = 0.02) |
| WISC: Working memory Index | 1.4 [0.12, 2.68] (p = 0.032) | 1.36 [-0.2, 2.91] (p = 0.089) | 0.43 [-1.44, 2.29] (p = 0.654) |
| **Duration of Breastfeeding *** | **Estimate [95% Cl] p-value** | **Estimate [95% Cl] p-value** | **Estimate [95% Cl] p-value** |
| Bayley-III Composite Score | -0.04 [-1.43, 1.35] (p = 0.955) | -0.4 [-1.44, 0.65] (p = 0.458) | -0.58 [-2.21, 1.06] (p = 0.491) |
| WISC: Full Scale Intelligence Quotient | -0.22 [-1.78, 1.35] (p = 0.785) | 0.82 [-0.56, 2.2] (p = 0.243) | 0.81 [-1.2, 2.82] (p = 0.43) |
| WISC: General Index | -0.24 [-2.16, 1.69] (p = 0.81) | 1.09 [-0.5, 2.69] (p = 0.181) | 0.77 [-1.62, 3.16] (p = 0.527) |
| WISC: Verbal comprehension Index | 0.61 [-1.22, 2.44] (p = 0.513) | 1.49 [-0.08, 3.05] (p = 0.064) | 0.63 [-1.67, 2.93] (p = 0.591) |
| WISC: Perceptual reasoning Index | -0.84 [-3.09, 1.41] (p = 0.464) | 0.31 [-1.58, 2.19] (p = 0.75) | 0.41 [-2.43, 3.25] (p = 0.777) |
| WISC: Processing speed Index | -0.17 [-1.83, 1.48] (p = 0.836) | 0.29 [-0.99, 1.56] (p = 0.662) | 0.9 [-1.1, 2.9] (p = 0.378) |
| WISC: Working memory Index | 0.09 [-1.42, 1.59] (p = 0.912) | 0.27 [-1.12, 1.66] (p = 0.705) | 0.28 [-1.72, 2.27] (p = 0.786) |

**S6 Table: Sex-Stratified Linear Regression Modelling of Dietary Exposures and Cognitive Outcomes.** This table presents the results of sex-stratified linear regression analyses assessing the associations between dietary exposures and cognitive outcomes; specifically, the Bayleys-III Composite score at 2.5 years and WISC at 10 years. The estimates are interpreted as the effect of a 1 standard deviation increase of pregnancy dietary pattern metabolite scores and breastfeeding duration. The associations are presented separately for females and males, with the interaction effects with child sex included.

*** Note breastfeeding is log-transformed and z-scored, thus estimates are interpreted as per 1 SD change.**
