# Supplementary material for: Maternal dietary patterns, breastfeeding duration, and their association with child cognitive function and head circumference growth: A prospective mother–child cohort study
Source: PLoS Med. 2025 Apr 10;22(4):e1004454. doi: 10.1371/journal.pmed.1004454 (PMC11984734; doi:10.1371/journal.pmed.1004454)
Supplement: S7 Table — (DOCX) [file pmed.1004454.s007.docx]

| **Western Dietary Pattern Metabolite Score Correlation Matrix** | **Maternal - 24 Weeks Gestation** | **Maternal - 1 week Postpartum** | **Child - 6 months** | **Child - 18 months** | **Child - 6 years** |
| --- | --- | --- | --- | --- | --- |
| **Maternal - 24 Weeks Gestation** | **1** | 0,68 | 0,42 | 0,46 | 0,33 |
| **Maternal - 1 week Postpartum** | 0,68 | **1** | 0,38 | 0,40 | 0,27 |
| **Child - 6 months** | 0,42 | 0,38 | **1** | 0,3 | 0,21 |
| **Child - 18 months** | 0,46 | 0,40 | 0,30 | **1** | 0,39 |
| **Child - 6 years** | 0,33 | 0,27 | 0,21 | 0,39 | **1** |
| **Varied Dietary Pattern Metabolite Score Correlation Matrix** | **Maternal - 24 Weeks Gestation** | **Maternal - 1 week Postpartum** | **Child - 6 months** | **Child - 18 months** | **Child - 6 years** |
| **Maternal - 24 Weeks Gestation** | **1** | 0,51 | 0,01 | 0,23 | 0,24 |
| **Maternal - 1 week Postpartum** | 0,51 | **1** | 0,02 | 0,26 | 0,24 |
| **Child - 6 months** | 0,01 | 0,02 | **1** | 0,12 | 0,17 |
| **Child - 18 months** | 0,23 | 0,26 | 0,12 | **1** | 0,19 |
| **Child - 6 years** | 0,24 | 0,24 | 0,17 | 0,19 | **1** |

**S7 Table: Correlation Coefficients between the Western Dietary Pattern Scores for Maternal Blood Metabolomes and Child Blood Metabolomes.** This table presents the correlation coefficients between the Western dietary pattern scores for maternal blood metabolomes (24 weeks pregnancy, 1 week postpartum) and child blood metabolomes (6 months, 18 months and 6 years). It provides a comprehensive overview of the correlations, highlighting the relationship between maternal and child metabolite scores at different time points.
