# Supplementary material for: Maternal dietary patterns, breastfeeding duration, and their association with child cognitive function and head circumference growth: A prospective mother–child cohort study
Source: PLoS Med. 2025 Apr 10;22(4):e1004454. doi: 10.1371/journal.pmed.1004454 (PMC11984734; doi:10.1371/journal.pmed.1004454)
Supplement: S8 Table — (DOCX) [file pmed.1004454.s008.docx]

|  | **Univariate Model** | **Multivariable Model** | **Multivariable with PRS** |
| --- | --- | --- | --- |
| **Latent Class Trajectory Model** | **Estimate [95% Cl] p-value** | **Estimate [95% Cl] p-value** | **Estimate [95% Cl] p-value** |
| WISC-IV: General Ability Index | 6.46 [3.83, 9.08] (p <0.001) | 4.81 [2.23, 7.40] (p = <0.001) | 3.88 [1.32, 6.45] (p = 0.003) |
| WISC-IV: Verbal comprehension Index | 4.98 [2.44, 7.52] (p <0.001) | 3.51 [0.98, 6.04] (p = 0.007) | 2.77 [0.25, 5.28] (p = 0.031) |
| WISC-IV: Perceptual reasoning Index | 6.18 [3.10, 9.25] (p <0.001) | 4.77 [1.69, 7.86] (p = 0.003) | 3.87 [0.77, 6.96] (p = 0.015) |
| WISC-IV: Processing speed Index | 0.26 [-1.91, 2.44] (p = 0.813) | 0.14 [-2.07, 2.35] (p = 0.901) | -0.28 [-2.52, 1.96] (p = 0.807) |
| WISC-IV: Working memory Core Index | 2.59 [0.40, 4.78] (p = 0.021) | 1.27 [-0.93, 3.47] (p = 0.259) | 0.59 [-1.61, 2.80] (p = 0.598) |
| **Linear Mixed Model (Slope)** | **Estimate [95% Cl] p-value** | **Estimate [95% Cl] p-value** | **Estimate [95% Cl] p-value** |
| WISC-IV: General Ability Index | 2.60 [1.49, 3.71] (p <0.001) | 1.72 [0.62, 2.83] (p = 0.002) | 1.42 [0.33, 2.51] (p = 0.011) |
| WISC-IV: Verbal comprehension Index | 2.04 [0.96, 3.11] (p <0.001) | 1.20 [0.12, 2.29] (p = 0.03) | 0.97 [-0.10, 2.04] (p = 0.076) |
| WISC-IV: Perceptual reasoning Index | 2.62 [1.33, 3.92] (p <0.001) | 1.91 [0.60, 3.23] (p = 0.005) | 1.61 [0.30, 2.93] (p = 0.016) |
| WISC-IV: Processing speed Index | 0.36 [-0.56, 1.27] (p = 0.443) | 0.24 [-0.70, 1.18] (p = 0.62) | 0.09 [-0.86, 1.04] (p = 0.847) |
| WISC-IV: Working memory Index | 1.01 [0.08, 1.94] (p = 0.033) | 0.27 [-0.67, 1.21] (p = 0.571) | 0.05 [-0.89, 0.99] (p = 0.919) |
| **Linear Mixed Model (Intercept)** | **Estimate [95% Cl] p-value** | **Estimate [95% Cl] p-value** | **Estimate [95% Cl] p-value** |
| WISC-IV: General Ability Index | 2.07 [0.90, 3.24] (p = 0.001) | 1.35 [0.21, 2.49] (p = 0.021) | 1.02 [-0.11, 2.15] (p = 0.076) |
| WISC-IV: Verbal comprehension Index | 1.61 [0.48, 2.75] (p = 0.005) | 0.96 [-0.16, 2.07] (p = 0.094) | 0.71 [-0.40, 1.82] (p = 0.211) |
| WISC-IV: Perceptual reasoning Index | 1.97 [0.61, 3.33] (p = 0.005) | 1.37 [0.02, 2.72] (p = 0.047) | 1.03 [-0.32, 2.38] (p = 0.134) |
| WISC-IV: Processing speed Index | 0.57 [-0.38, 1.53] (p = 0.241) | 0.55 [-0.41, 1.51] (p = 0.265) | 0.38 [-0.59, 1.36] (p = 0.444) |
| WISC-IV: Working memory Index | 1.78 [0.82, 2.74] (p <0.001) | 1.28 [0.33, 2.24] (p = 0.009) | 1.05 [0.09, 2.01] (p = 0.033) |

**S8 Table: Western Dietary Pattern Metabolite Score during Pregnancy and Head Circumference Growth: Latent Class and Linear Mixed Model Results.** This table presents the results of linear regression analyses assessing the associations between a Western dietary pattern metabolite score during pregnancy and head circumference growth, as determined by latent class trajectory modelling and linear mixed modelling. The latent class trajectory model estimates compare the “Increasing” (26%) class to the “Reference” (74%) class. Estimates for linear mixed model slope and intercept are interpreted as the effect of 1 standard deviation within our population. The table provides both unadjusted and adjusted associations, with the latter controlling for potential confounders such as pre-pregnancy maternal body mass index, child sex, birth weight, gestational age, smoking during pregnancy, antibiotic use during pregnancy, pre-eclampsia, household income at birth, maternal education level at birth, maternal age at birth and breastfeeding duration. Further adjustments include maternal and child polygenic risk score for intelligence, and child head circumference polygenic risk scores.
