# Supplementary material for: Maternal dietary patterns, breastfeeding duration, and their association with child cognitive function and head circumference growth: A prospective mother–child cohort study
Source: PLoS Med. 2025 Apr 10;22(4):e1004454. doi: 10.1371/journal.pmed.1004454 (PMC11984734; doi:10.1371/journal.pmed.1004454)
Supplement: S9 Table — (DOCX) [file pmed.1004454.s009.docx]

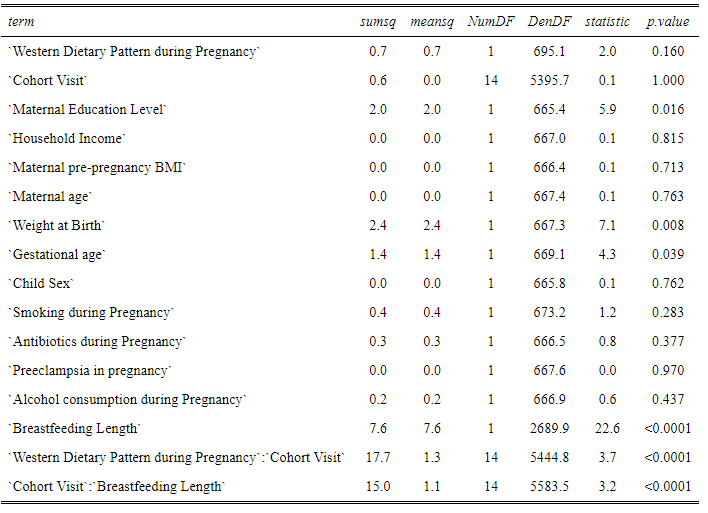


**S9 Table. ANOVA Table for the Multivariable Linear Mixed Model of Western Dietary Pattern Metabolite Score during Pregnancy and Breastfeeding Duration**. This table presents the ANOVA table for the multivariable linear mixed model, showing the interaction terms over time for the Western dietary pattern metabolite score during pregnancy and breastfeeding duration with calibrated head circumference. The coefficients indicate that a Western dietary pattern metabolite score during pregnancy (p<0.001) is associated with children with decreasing head circumference over time, whereas longer breastfeeding (p<0.001) was associated with increasing head circumference over time.
