# Supplementary material for: Maternal dietary patterns, breastfeeding duration, and their association with child cognitive function and head circumference growth: A prospective mother–child cohort study
Source: PLoS Med. 2025 Apr 10;22(4):e1004454. doi: 10.1371/journal.pmed.1004454 (PMC11984734; doi:10.1371/journal.pmed.1004454)
Supplement: S1 Fig — (DOCX) [file pmed.1004454.s010.docx]

**
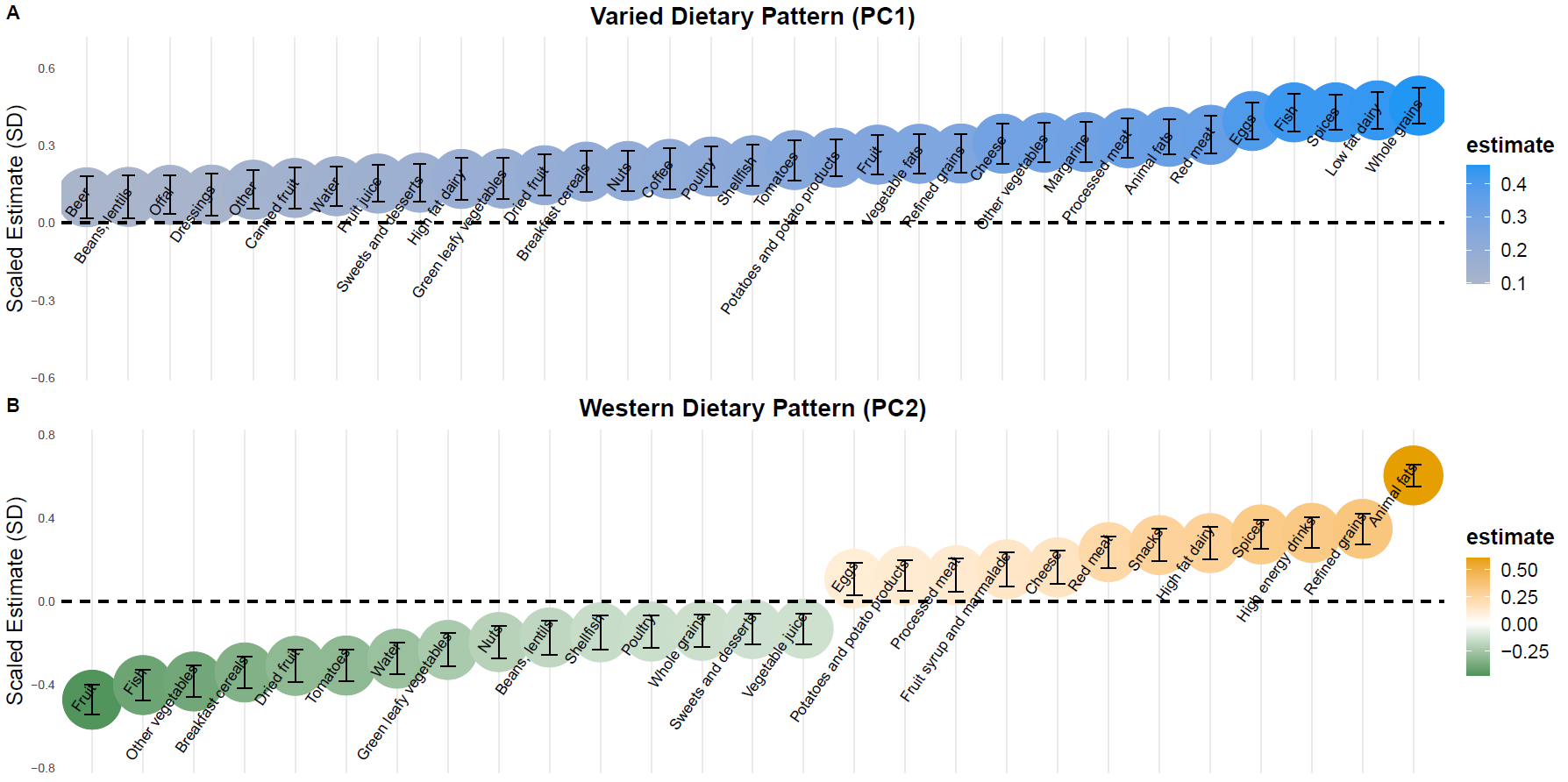
**

**S1 Fig. Principal Component Loadings for the Varied and Western Dietary Patterns.** This supplementary figure presents the principal component loadings for the Varied Dietary Pattern (Panel A) and Western Dietary Pattern (Panel B). The scaled estimates (standard deviations) illustrate the contribution of individual food groups to each dietary pattern. Positive loadings indicate higher consumption of a food group within the dietary pattern, while negative loadings reflect lower consumption.
