# Supplementary material for: Maternal dietary patterns, breastfeeding duration, and their association with child cognitive function and head circumference growth: A prospective mother–child cohort study
Source: PLoS Med. 2025 Apr 10;22(4):e1004454. doi: 10.1371/journal.pmed.1004454 (PMC11984734; doi:10.1371/journal.pmed.1004454)
Supplement: S2 Fig — (DOCX) [file pmed.1004454.s011.docx]

**
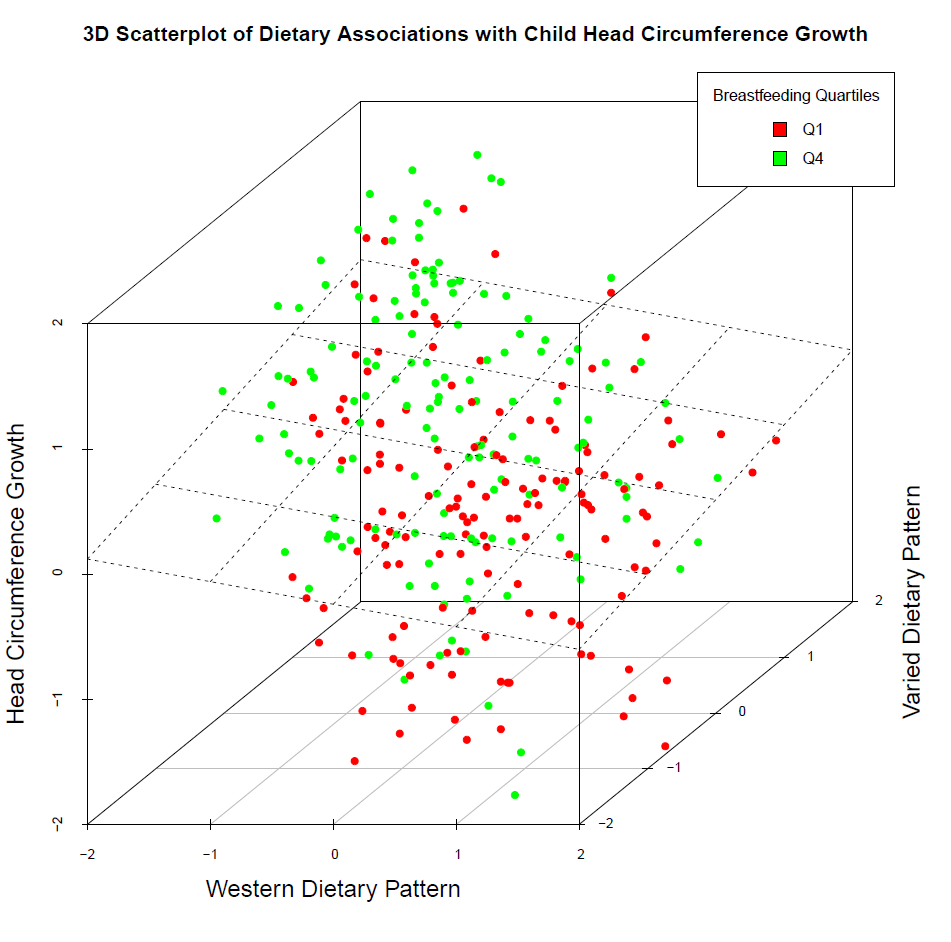
**

**S2 Fig. Three-Dimensional Scatterplot Illustrating the Associations Between Maternal Diet, Cognition, and Head Circumference Growth.** This supplementary figure presents a three-dimensional scatterplot that elucidates the intricate relationships between dietary patterns during pregnancy, breastfeeding duration, and head circumference growth. The X-axis represents the Western dietary pattern metabolite score during pregnancy (reflecting a 1 SD change in our population), the Z-axis represents the Varied dietary pattern (reflecting a 1 SD change in our population), and the Y-axis represents head circumference growth (derived from the slope of the linear mixed model, reflecting a 1 SD change in our population). Green points indicate mothers who breastfed the most (Q4), while red points indicate mothers who breastfed the least (Q1). The plane illustrates the relationship between the Western dietary pattern and child breastfeeding duration on head circumference growth.
