# Supplementary material for: Maternal dietary patterns, breastfeeding duration, and their association with child cognitive function and head circumference growth: A prospective mother–child cohort study
Source: PLoS Med. 2025 Apr 10;22(4):e1004454. doi: 10.1371/journal.pmed.1004454 (PMC11984734; doi:10.1371/journal.pmed.1004454)
Supplement: S3 Fig — (DOCX) [file pmed.1004454.s012.docx]

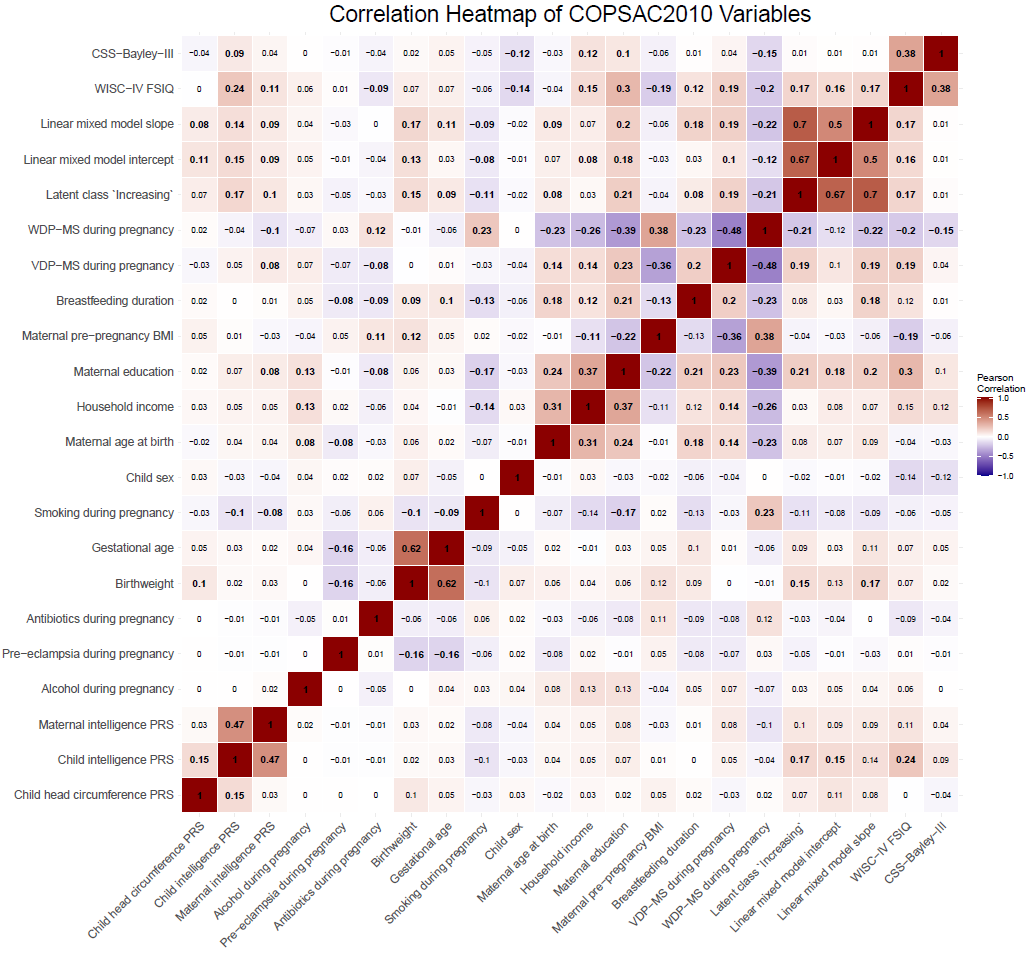


**S3 Fig. Heatmap of Correlations between Dietary Exposures, Outcomes, and Model Covariates.** This supplementary figure presents a comprehensive heatmap visualising the correlations between dietary exposures, outcomes, and model covariates. The linear mixed model parameters (slope and intercept) and latent class variables (Increasing vs Reference) included in the heatmap are derived from the longitudinal modelling of head circumference growth. The Varied dietary pattern metabolite score (VDP-MS) and Western dietary pattern metabolite score (WDP-MS) are derived from blood metabolome modelling of the principal components from pregnancy food frequency questionnaires.
