# Supplementary material for: Maternal dietary patterns, breastfeeding duration, and their association with child cognitive function and head circumference growth: A prospective mother–child cohort study
Source: PLoS Med. 2025 Apr 10;22(4):e1004454. doi: 10.1371/journal.pmed.1004454 (PMC11984734; doi:10.1371/journal.pmed.1004454)
Supplement: S4 Fig — (DOCX) [file pmed.1004454.s013.docx]

**
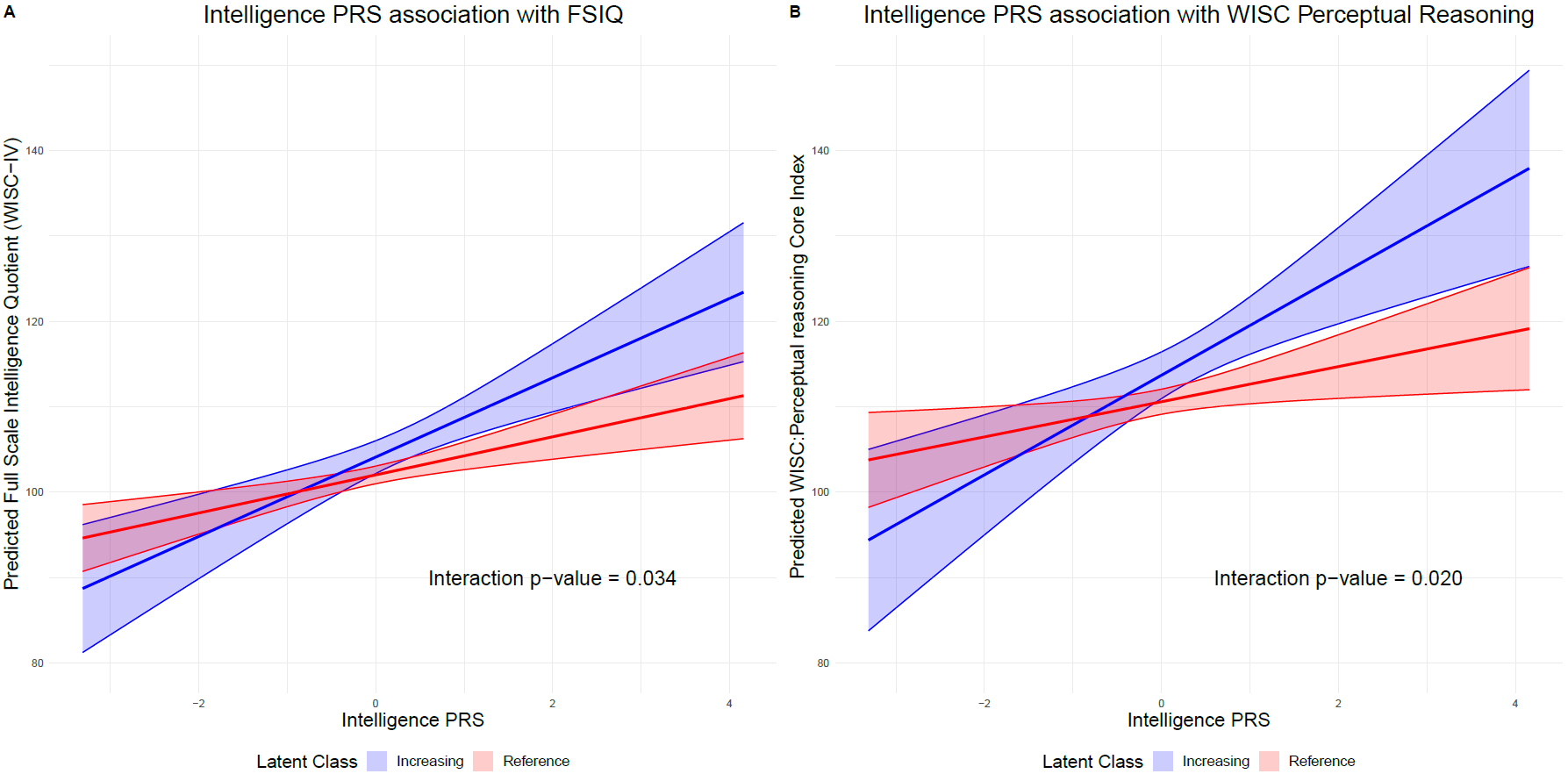
**

**S4 Fig. Modulating Effects of Genetics Scores for Head Circumference and Intelligence on the Association between Head Circumference and Cognition at 10 Years.** This figure presents the results of a sensitivity analysis assessing the modulating effects of genetics scores for head circumference and intelligence on the association between head circumference and cognition at 10 years. The figure illustrates the significant and positive effect modulation of the child intelligence and head circumference latent classes, suggesting that children with higher genetics for intelligence, and those adhering to the 'Increasing' latent class, benefit from a stronger association between head circumference and cognition.
