# Supplementary material for: Maternal dietary patterns, breastfeeding duration, and their association with child cognitive function and head circumference growth: A prospective mother–child cohort study
Source: PLoS Med. 2025 Apr 10;22(4):e1004454. doi: 10.1371/journal.pmed.1004454 (PMC11984734; doi:10.1371/journal.pmed.1004454)
Supplement: S5 Fig — (DOCX) [file pmed.1004454.s014.docx]

**
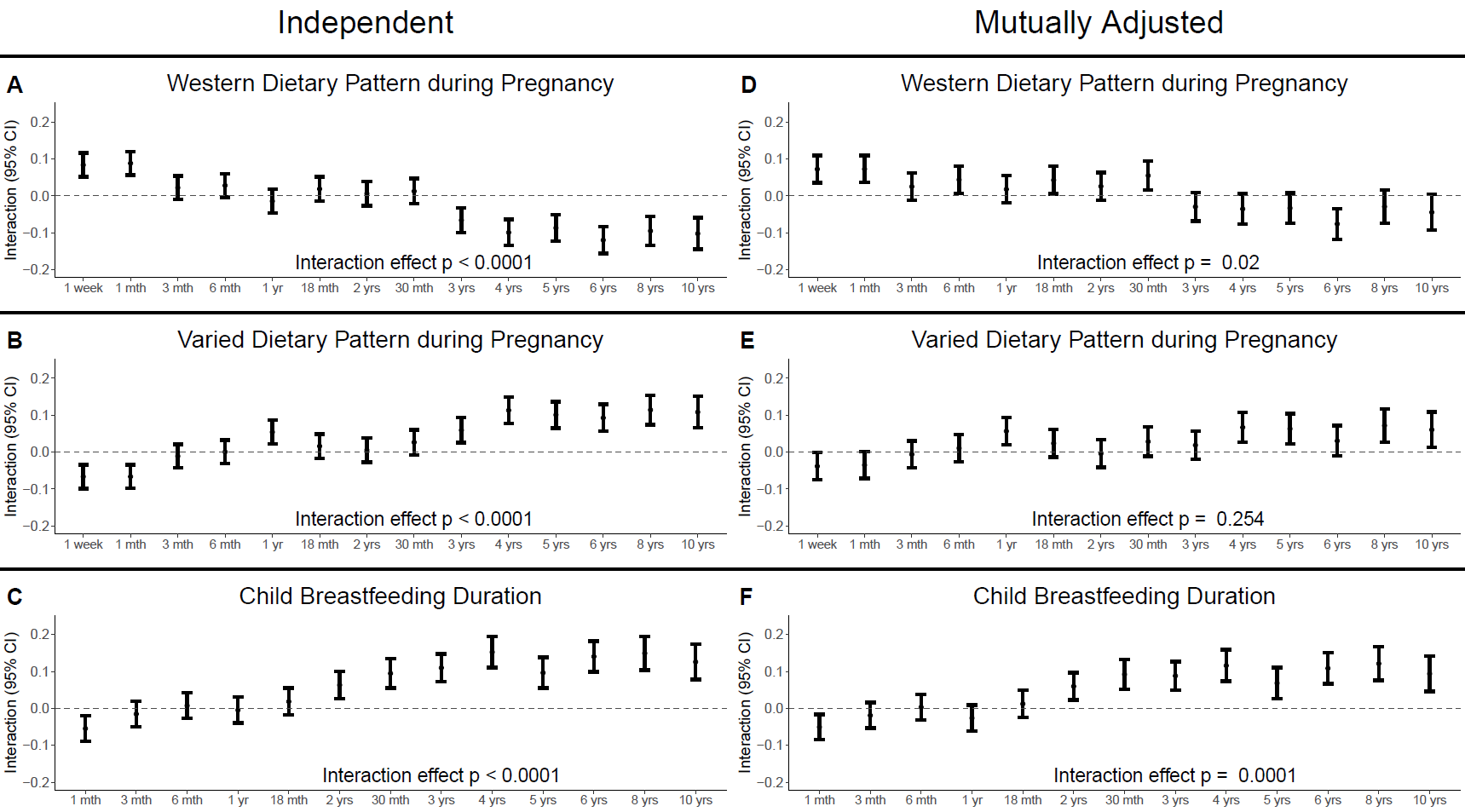
**

**S5 Fig. Independent and Mutual Interaction Term Effects of Dietary Exposures.** This supplementary figure presents the independent Western dietary pattern during pregnancy, Varied dietary pattern during pregnancy, and child breastfeeding duration interaction term effects in multivariable models at each clinical visit. The figure also shows the respective models after mutual adjustment for the Western dietary pattern during pregnancy, Varied dietary pattern during pregnancy, and child breastfeeding duration. The figure illustrates that only the Western dietary pattern and breastfeeding duration remained significant after mutual adjustment.
