# Supplementary material for: Maternal dietary patterns, breastfeeding duration, and their association with child cognitive function and head circumference growth: A prospective mother–child cohort study
Source: PLoS Med. 2025 Apr 10;22(4):e1004454. doi: 10.1371/journal.pmed.1004454 (PMC11984734; doi:10.1371/journal.pmed.1004454)
Supplement: S6 Fig — (DOCX) [file pmed.1004454.s015.docx]

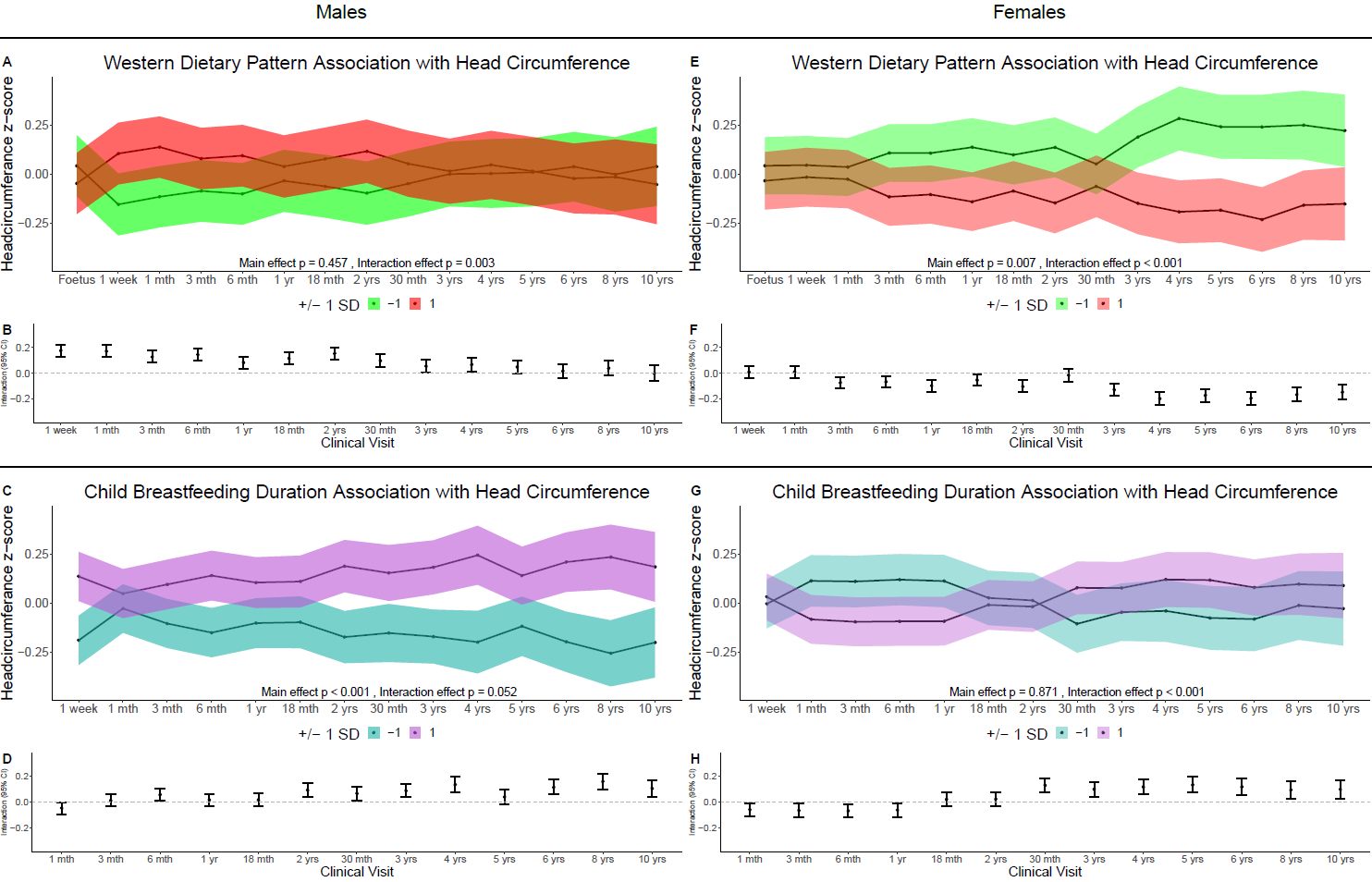


**S6 Fig. Sex Stratified Associations of a Pregnancy Western Dietary Pattern and Breastfeeding Duration on Longitudinal Measures of Head Circumference in a Multivariable Linear Mixed Model.** This supplementary figure presents the sex-stratified effects of a pregnancy Western dietary pattern and breastfeeding duration on longitudinal measures of head circumference (+/- 1 SD). Panels A-D represent males and Panels E-H represent females. The figure illustrates the predicted head circumference with 95% confidence limits for the Western dietary pattern metabolite score during pregnancy and breastfeeding duration, as well as the interaction term effects at each clinical visit.
