# Supplementary material for: Maternal dietary patterns, breastfeeding duration, and their association with child cognitive function and head circumference growth: A prospective mother–child cohort study
Source: PLoS Med. 2025 Apr 10;22(4):e1004454. doi: 10.1371/journal.pmed.1004454 (PMC11984734; doi:10.1371/journal.pmed.1004454)
Supplement: S7 Fig — (DOCX) [file pmed.1004454.s016.docx]

**
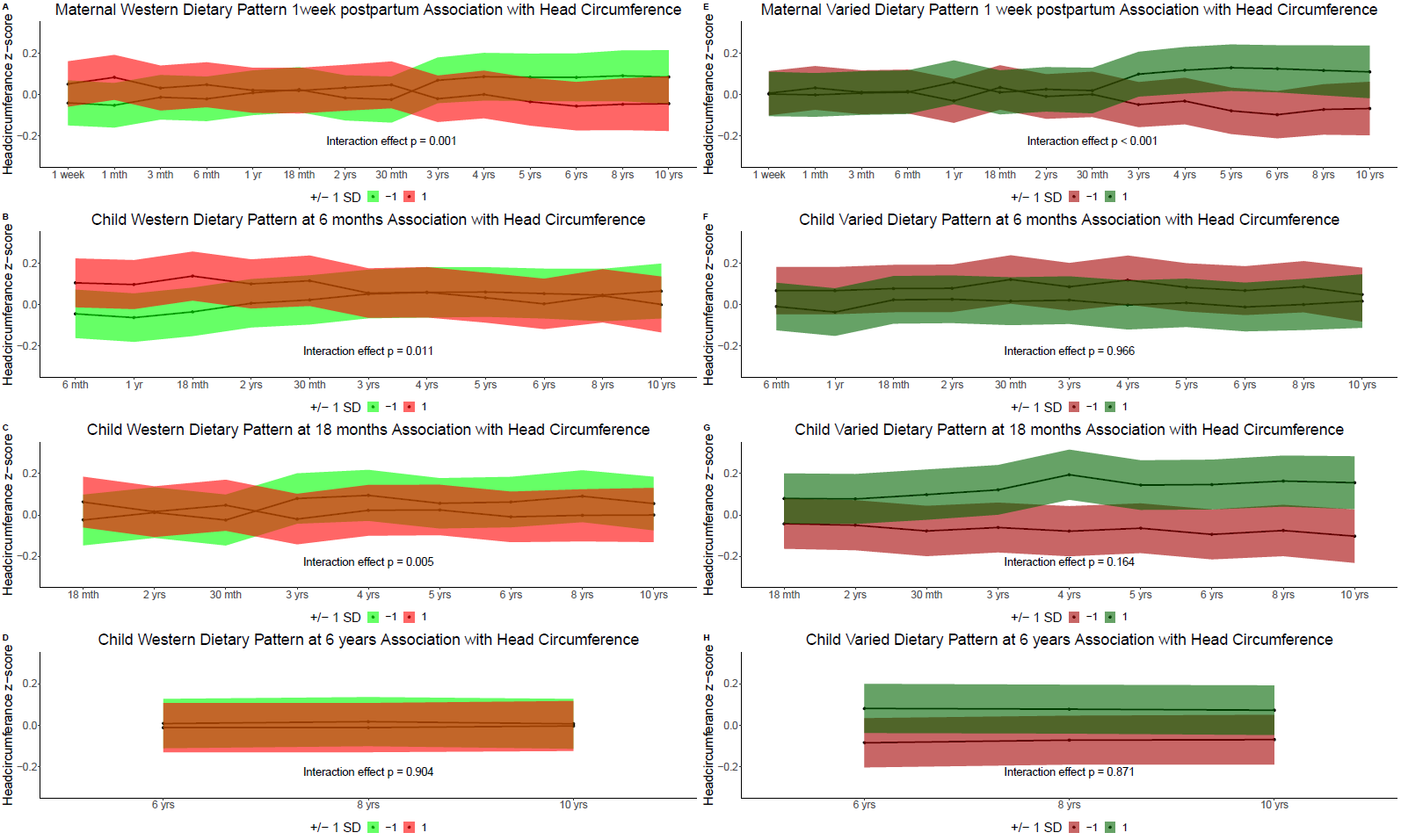
**

**S7 Fig. Independent Effects of the Western and Varied Dietary Metabolite Scores for Mothers 1 Week Postpartum and Children's Metabolome at Various Time Points on Head Circumference.** This supplementary figure presents the independent effects of the Western dietary metabolite scores (A-D) and Varied dietary pattern metabolites scores (E-H) for mothers 1 week postpartum and children's metabolome at 6 months, 18 months, and 6 years on head circumference (+/- 1 SD). The figure shows the predicted head circumference with 95% confidence limits for the dietary pattern metabolite scores.
