# Supplementary material for: Maternal dietary patterns, breastfeeding duration, and their association with child cognitive function and head circumference growth: A prospective mother–child cohort study
Source: PLoS Med. 2025 Apr 10;22(4):e1004454. doi: 10.1371/journal.pmed.1004454 (PMC11984734; doi:10.1371/journal.pmed.1004454)
Supplement: S8 Fig — (DOCX) [file pmed.1004454.s017.docx]

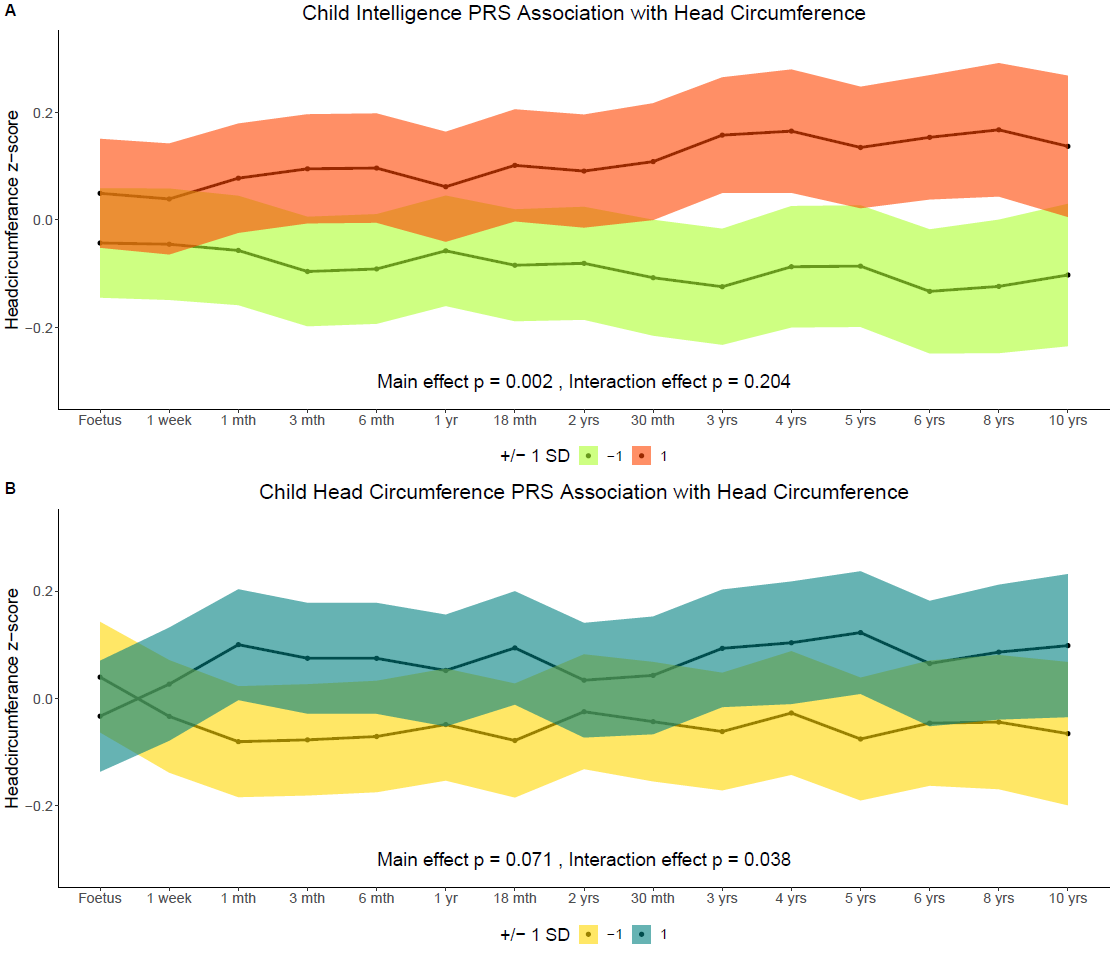


**S8 Fig. Effect of Child Intelligence and Head Circumference Polygenic Risk Score on Longitudinal Measures of Head Circumference.** This figure presents the effect of child intelligence and head circumference polygenic risk score on longitudinal measures of head circumference (+/- 1 SD). The figure illustrates the direct influence of genetic propensity for intelligence (panel A), and head circumference (panel B), on overall head circumference and the time-dependent association of the head circumference polygenic risk score on head growth.
