# Supplementary material for: Maternal dietary patterns, breastfeeding duration, and their association with child cognitive function and head circumference growth: A prospective mother–child cohort study
Source: PLoS Med. 2025 Apr 10;22(4):e1004454. doi: 10.1371/journal.pmed.1004454 (PMC11984734; doi:10.1371/journal.pmed.1004454)
Supplement: S1 File — (PDF) [file pmed.1004454.s019.pdf]

SUPPLEMENTARY METHODS

Breastfeeding Duration Data Normalisation and Modelling

Regarding breastfeeding duration, to normalise the right-skewed data for analysis (*Figure 1F*), we imputed 1-day for 0-day instances, followed by a log-transformation and scaling in our main analysis. For longitudinal analysis, we considered the number of days breastfeeding up until that visit of measurement. To address multicollinearity due to low variance at the foetal time point for head circumference, we imputed data from the subsequent time point (1 week).

Blood Metabolome

Untargeted plasma metabolomics data was collected from mothers at mid-pregnancy (24 weeks gestation) and one week postpartum, as well as from children at the ages of 6 months, 18 months, and 6 years. Blood samples were taken using an EDTA tube during research clinic visits and centrifuged at 4000 rpm for 10 minutes to obtain plasma. The supernatant was stored at −80 °C for future analysis. Metabolon, Inc. (NC, USA) performed the untargeted metabolomic analysis.

Sample preparation was automated using the MicroLab STAR® system from Hamilton Company. Recovery standards were added to each sample for quality control (QC) before methanol extraction of metabolites. This process involved vigorous shaking for 2 minutes with a Glen Mills GenoGrinder 2000, followed by centrifugation to precipitate proteins. The resulting extract was divided into four aliquots for analysis on four distinct LC-MS/MS platforms. The aliquots were dried using a TurboVap® (Zymark) to evaporate the solvent and stored under nitrogen overnight before LC-MS/MS preparation.

Analysis was conducted using an ACQUITY Ultra-Performance Liquid Chromatography (UPLC) system by Waters, Milford, USA, coupled with a Q Exactive™ Hybrid Quadrupole-Orbitrap™ mass spectrometer, featuring a heated electrospray ionisation (HESI-II) source, from ThermoFisher Scientific, Waltham, Massachusetts, USA. Specific solvent mixes were used for the four LC methods: two reverse phase UPLC-ESI(+) MS/MS methods for hydrophilic and hydrophobic molecules, one reverse phase UPLC-(−) MS/MS method, and one HILIC/UPLC-(−) MS/MS method. The mass spectrometry alternated between full scan MS and data-dependent MSn scans with dynamic exclusion, covering a range from 70 to 1000 m/z for both ion modes.

Data collection and quality control included raw data extraction, peak identification, and QC procedures. Semi-quantification of samples was based on the area-under-the-curve method. Further details are provided in our prior publications (65). Data preprocessing excluded metabolites with more than 33% missingness. Remaining missing data was imputed using random forest imputation (missForest package, v1.5) (66), and the metabolome data was log-transformed, centred, and scaled before analysis.

For mothers at mid-pregnancy (24 weeks gestation), a total of 760 annotated metabolites were analysed. Among these, 744 metabolites overlapped between the 24-week gestation and one week postpartum, 516 with the 6-month data, 707 with the 18-month data, and 540 with the 6-year data.

Modelling of Dietary Pattern Metabolite Scores

We utilised the caret package (v6.0.90) to establish Western and Varied dietary pattern metabolite scores via sparse partial least squares regression on the metabolomics datasets, with the FFQ-derived pregnancy Western and Varied dietary patterns as the response variable. Individual models were created to predict Western and Varied dietary pattern metabolite scores at other timepoints using the subset of overlapping metabolites (see `Blood Metabolome`). To enhance interpretability and minimise the risk of overfitting, we employed single-component models with cross-validated predictions (repeated cross-validation, number of segments=5, repeats=10). After evaluating models with varying sparsity (incremented by 0.1 from 0 to 1), we selected the model with the lowest root-mean-square error for cross-validation (RMSECV) as our optimal model.
