# Supplementary material for: Maternal dietary patterns, breastfeeding duration, and their association with child cognitive function and head circumference growth: A prospective mother–child cohort study
Source: PLoS Med. 2025 Apr 10;22(4):e1004454. doi: 10.1371/journal.pmed.1004454 (PMC11984734; doi:10.1371/journal.pmed.1004454)
Supplement: Attachment — Submitted filename: Reviewer Point by Point Response.pdf [file pmed.1004454.s021.pdf]

PLOS Medicine Point-by-point Response

Dietary Patterns in Pregnancy and Child Breastfeeding Duration are Associated with Cognitive Function and Head Circumference Growth in Childhood

David Horner MD, MSc[1], Jens Richardt Møllegaard Jepsen, MSc, PhD[2+3], Bo Chawes, MD, PhD, DMSc[1], Rebecca Vinding[1], MD, PhD, Julie B. Rosenberg, MD[1+2+4], Parisa Mohammadzadeh, MD[1+2+4], Yang Luo PhD[21], Birgitte Fagerlund, PhD[2+5], Trine Flensborg-Madsen, PhD DMSci[6], Thomas Ragnar Wood, MD, PhD[7], Janine F. Felix, PhD[8+9], Louise Monnerup, RN[1], Birte Y. Glenthøj, MD DMSc[2+4], Klaus Bønnelykke, MD PhD[1], Bjørn H. Ebdrup*****, MD, PhD[2+4], Jakob Stokholm*****, MD, PhD[1+10], Morten Rasmussen***** PhD[1+10]

Comments from the academic editor:

The academic editor supported the decision to move forward to a major revision outcome. He commented that he thought your manuscript was ‘overall well written and balanced’. However, he asked that the authors add a bit more to their main text about residual confounding and risk of spurious associations.

Authors Response #1

Thank you for reviewing our manuscript and providing your input and expertise. Based on your feedback we have included the following sentence in the main text, located in the discussion section:

Line 476: “*Regarding the limitations of our observational study, despite extensive adjustments for genetic and phenotypic factors, residual confounding due to unmeasured variables related to maternal or child phenotypes cannot be entirely excluded, and some associations may be spurious; however, supplemental analysis adjusting for maternal genetic risk for intelligence did not change our findings.*”

Reviewer #1:

"Dietary Patterns in Pregnancy and Child Breastfeeding Duration are Associated with Cognitive Function and Head Circumference Growth in Childhood" studies how maternal dietary patterns (defined as "varied"/"Western") during pregnancy and duration of breastfeeding, are associated with child head circumference and cognitive outcomes, on 700 mother-child pairs from Denmark. It was concluded that a Western dietary pattern is associated with lower cognitive scores/decreased head circumference growth, as opposed to a varied dietary pattern.

Some issues might be considered:

1. In Line 163, it is stated that the first principal component reflected a varied dietary pattern (44.3% of participants), and the second component reflected a Western dietary pattern (10.7%). Details about how the principal components were analysed/obtained, and how each participant was eventually assigned to varied/Western dietary pattern (from the metabolite scores & questionnaire, and the meaning of the dietary pattern axes in Supplementary Figure S1 etc.), should be provided given its centrality to the study. While Figure 1 shows some information, it remains unclear as to how a participant would be assigned to varied/Western.

Authors Response #2

Thank you for reviewing our manuscript and your comments.

We noted that all three reviewers asked for more information and clarification regarding the formation of the two pregnancy dietary patterns (`Varied dietary pattern` and `Western dietary pattern`) and we acknowledge that our Methods section in the main text of the original manuscript would benefit greatly from this additional information. We appreciate the importance of this, given that these dietary patterns are the main exposures of interest in our work. We have therefore conducted a major revision of our Methods section, whereby we have incorporated and bolstered information which was originally found in the "Supplementary Methods" section, to the main text, to provide this clarification.

With regard to your comments of assignment of dietary patterns, due to the nature of principal component analysis, individuals were not "assigned" but were rather provided a numerical score, or a weighting which for clarity we have z-scored (see histogram below of the 684 mothers with metabolite score data).

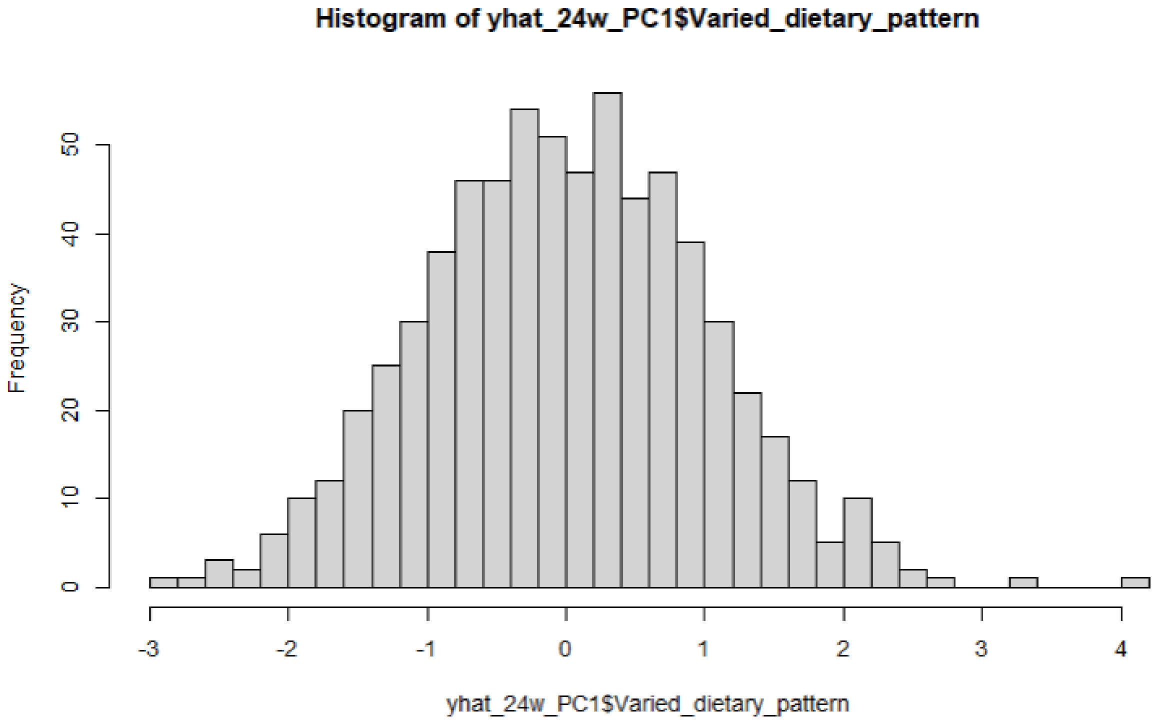

As such, an individual with a high scoring for PC1 (e.g. 2), would be considered as having a more "Varied dietary pattern", and individuals with a lower scoring for PC1 (e.g. -2) would be considered as having a low "Varied dietary pattern". This score is z-score to allow for appropriate interpretation of model effect estimates (i.e. per 1SD in our population). We hope that the specific changes we have outlined below based on your comments in our updated Methods section provide further clarity.

Based on your comments we have made the following changes to our manuscript:

> Further detailed how the principal components were derived from pregnancy FFQs and used to make the quantitative metabolite scores.

Line 193 "*Principal component analysis (PCA) identified dietary patterns based on calculated estimates of energy, macronutrients, and micronutrients derived from pregnancy food frequency questionnaires. The first principal component reflected a "Varied dietary pattern" (44.3%), while the second reflected a "Western dietary pattern" (10.7%), these dietary patterns were used to derive the metabolite scores in the sPLS models."*

> Clarified the meaning of the dietary axes in Supplementary Figure 1

"The X-axis represents the Western dietary pattern metabolite score during pregnancy (reflecting a 1 SD change in our population), the Z-axis represents the Varied dietary pattern (reflecting a 1 SD change in our population), and the Y-axis represents head circumference growth (derived from the slope of the linear mixed model, reflecting a 1 SD change in our population)."

> Clarified the use z-scoring of our exposure data, which facilitates accurate interpretation of our graphical data and modelling estimates

Line 203: "*Metabolite scores and breastfeeding length were internally z-scored at each timepoint, thus reflecting a change of 1 standard deviation within the population.*"

2. Moreover, it might be discussed as to how only varied/Western dietary patterns were defined - other dietary patterns (e.g. vegan, vegetarian, etc.) would seem plausible too.

Authors Response #3

Thank you for your comment. Our work specifically aimed to investigate dietary patterns associated with nutrient content, as discussed in our introduction section, where this approach is highlighted as a plausible mechanism for capturing the synergistic effects of nutrients on neurodevelopment, rather than focusing on predefined food-based patterns such as vegan or vegetarian diets.This approach is supported by a recent meta-analysis across 123 studies, which demonstrated the reliability of using nutrient-derived patterns from food frequency questionnaires (PMID 33874970).

Our decision to focus on nutrient-based patterns stems from the rationale that nutrients provide direct biological insights and are less likely to be confounded by cultural or regional dietary habits, thereby enhancing their translatability across diverse populations. The "Varied" and "Western" dietary patterns were data-driven, utilising validated food frequency questionnaire (FFQ) data analysed through principal component analysis (PCA). These two patterns were selected as they accounted for the largest variance in nutrient consumption within our population. We agree that pursuing alternative dietary habits, such as vegan or vegetarian diets in pregnancy, are plausible and valuable areas of exploration. However, such diets fall outside the remit of our current analysis, which focused on nutrient-driven dietary patterns. We hope this explanation clarifies the rationale for our chosen dietary pattern definitions.

3. From the covariates presented in the section beginning Line 214, possibly relevant variables such as ethnicity and parental (including paternal) intelligence/IQ (this perhaps estimated with maternal education as a proxy) appear to be missing. Authors might discuss the most relevant variables affecting child head circumference/cognitive outcomes from prior work, since it is currently only briefly acknowledged that heritability estimates for intelligence are high (if lower in childhood), but without context of their relative impact.

Authors Response #4

Thank you for your comment regarding our selection of covariates. We have carefully considered your feedback and have updated the manuscript to include additional references supporting the rationale for the chosen covariates. Specifically, we aimed to focus on the most relevant variables influencing child head circumference and cognitive outcomes, while ensuring that our analysis remained robust and interpretable.

Line 259: "*Potential confounders were identified with a directed acyclic graph and existing literature (13,44–47).*"

Regarding the lack of ethnicity as a covariate in our analysis. We would highlight that our population sample is 96% caucasian race (Table 1), and therefore we do not usually adjust for race as a covariate, as we lack the variance in our dataset. Additionally we would also highlight previous concerns shared by experts in specifically adjusting for ethnicity with regard to head circumference (https://www.bmj.com/content/354/bmj.i4721/rr-1). You are also correct that we do not adjust for parental IQ/intelligence, and in assuming that we use maternal education as a proxy for capturing this, as we lack parental intelligence/IQ in our dataset. We believe that our limitations statement that we have included in the discussion section sufficiently incorporates this by referring to potential remaining residual confounding.

Line 476 "*Regarding the limitations of our observational study, despite extensive adjustments for genetic and phenotypic factors, residual confounding due to unmeasured variables related to maternal or child phenotypes cannot be entirely excluded…* "

We believe these additional references strengthen the manuscript by providing additional clarity and context for our covariate selection and addressing the key points raised in your feedback.

4. For Supplementary Figure S2, it might be considered to briefly explain the linear mixed model parameter & latent class variables - which outcome were they for?

5. Related to the above, it would be interesting to consider the raw correlations between the two dietary patterns and the other variables, if possible.

Authors Response #5

Thank you for your comments regarding our Supplementary Figure 2. We appreciate that our axis labelling of "Linear mixed model intercept", "Linear mixed model slope" and "Latent class `Increasing`" may provide some confusion without appropriate context. We have therefore updated the figure text, to highlight that these variables refer to modelling of longitudinal head circumference growth for enhanced clarity. For additional clarity we have included that "VDP-MS" and "WDP-MS" are shortened for Varied dietary pattern and Western dietary pattern metabolite scores (addressing your point 5.).

Updated Figure Text:

Supplementary Figure S2. Heatmap of Correlations between Dietary Exposures, Outcomes, and Model Covariates. *This supplementary figure presents a comprehensive heatmap visualising the correlations between dietary exposures, outcomes, and model covariates. The linear mixed model parameters (slope and intercept) and latent class variables (Increasing vs Reference) included in the heatmap are derived from the longitudinal modelling of head circumference growth. The Varied dietary pattern metabolite score (VDP-MS) and Western dietary pattern metabolite score (WDP-MS) are derived from blood metabolome modelling of the principal components from pregnancy food frequency questionnaires.*

6. While it is stated that 700 mother-child pairs were involved, Table 1 (n=684) and Supplementary Tables S1 & S2 (n=695) give different cohort sizes. This might be clarified, possibly alongside the inclusion of a flowchart detailing any exclusion steps.

Authors Response #6

Thank you for your comment regarding our cohort size, and sample sizes included in our analysis.

Whilst 700 mother-child pairs are included in the COPSAC2010 cohort, we opted to remove twin-pairs to ensure independence of our findings (n=695).

Line 281 *"We removed the second twin child in twin pairs (n=5) from our analysis, since the exposures of interest were not independent."*

We have updated our `Baseline Characteristics and Dietary Exposures` section of our Results section to provide more clarity in the cohort numbers available in our subsequent analysis.

Line 290: "*Dietary exposures included dietary pattern metabolite scores derived from maternal blood metabolome data (available for 684 mothers) and breastfeeding duration data (available for 687 mother-child pairs). Outcomes assessed were cognition, evaluated in 627 (90.2%) children at 2.5 years and 586 children (84.3%) at 10 years, and head circumference growth, based on 9,501 measurements collected from 20 weeks gestation to 10 years (8.5% data missing). Each child in the main analysis contributed a median of 15 head circumference measurements (IQR 13-15).*"

We hope these steps we have taken help clarify the numbers included in our subsequent analysis. We again thank you for the time and efforts you have taken in reviewing our work.

Reviewer #2:

This study examined the associations between maternal dietary pattern during pregnancy and breastfeeding and children's cognitive development. Their results suggest that the Western dietary pattern during pregnancy was associated with lower cognitive scores at 2.5 y and less head circumference growth while a varied dietary pattern was associated with higher IQ at 10y and head circumference growth as a contributor. The study was well-conducted and the longitudinal nature of the study provided valuable novel evidence regarding the long-lasting influence of maternal diet on cognitive outcomes of offspring. Here are some questions:

Line 157-168: It was confusing how the metabolomics and FFQ data were combined together to generate a dietary pattern.

Authors Response #7

Thank you for reviewing our work, and for your positive comments regarding the quality of our work.

All 3 reviewers provided comments expressing confusion regarding our description of the FFQ-derived dietary patterns, and subsequent dietary pattern modelling. As such we have provided a major revision of the manuscript main text, where previously many of these methods were only described in the "Supplementary Methods". We appreciate the importance of accurately describing our main exposure in our work and hope that these changes provide clarity to you and the other two reviewers.

Updated Methods:

Line 188: "*Our main exposures included dietary pattern metabolite scores, assessed from global blood metabolomics profiles taken at 24 weeks gestation (HD4 platform, Metabolon, Inc. (NC, USA)), derived from sparse partial least squares (sPLS) modelling, and breastfeeding duration (length of breastfeeding in days). Dietary pattern metabolite scores were modelled on dietary patterns during pregnancy, as identified from a validated 360 item semi-quantitative food frequency questionnaire (FFQ) completed by mothers at recruitment (24 weeks gestation), with a one-month recall period (33,34). Principal component analysis (PCA) identified dietary patterns based on calculated estimates of energy, macronutrients, and micronutrients derived from pregnancy food frequency questionnaires. The first principal component reflected a "Varied dietary pattern" (44.3%), while the second reflected a "Western dietary pattern" (10.7%), these dietary patterns were used to derive the metabolite scores in the sPLS models. Breastfeeding duration, measured as the length of breastfeeding in days, was also considered as a dietary exposure. Further details regarding the blood metabolomics analysis, derivation of dietary pattern metabolite scores via sPLS modelling, and data normalisation procedures for breastfeeding duration can be found in the Supplementary Methods.*"

What was the evidence base that those metabolites were reflective of dietary exposure and how it was reconciled with the food frequency from FFQ?

Authors Response #8

Thank you for your comment. The use of blood metabolites as markers of dietary exposure is increasingly recognised and utilised in the literature as a robust quantitative approach to measure dietary intake (PMID 37804582). Untargeted plasma metabolomics provides an objective biochemical representation of dietary intake, complementing self-reported data from FFQs and addressing potential recall bias.

In this study, sparse partial least squares (sPLS) regression was used to align metabolomics data with dietary patterns derived from FFQs. This method ensures that the metabolite scores correspond to the Western and Varied dietary patterns identified through principal component analysis, creating a biochemical link to self-reported dietary habits.

This integration of biochemical and self-reported data provides a robust framework for exploring the relationship between diet and health outcomes. By using metabolomics to objectively validate dietary patterns, the study strengthens the reliability of its findings, linking subjective dietary behaviors with their measurable biochemical effects.

We have incorporated the following sentences in our discussion section to clarify and support our use of blood metabolomics:

Line 466: *"The increasing utilisation and validation of metabolomics for assessing dietary exposures underscores the growing importance of this method in enhancing the reliability and accuracy of dietary assessments (59). Accordingly, we have in previous work externally validated the Western dietary pattern metabolite score in an independent cohort (30)."*

For further convenience I have provided a figure, from an existing publication where we externally validated the same Western dietary pattern metabolite score used in this work, in an external American mother-child cohort, against pregnancy reported FFQ food items.

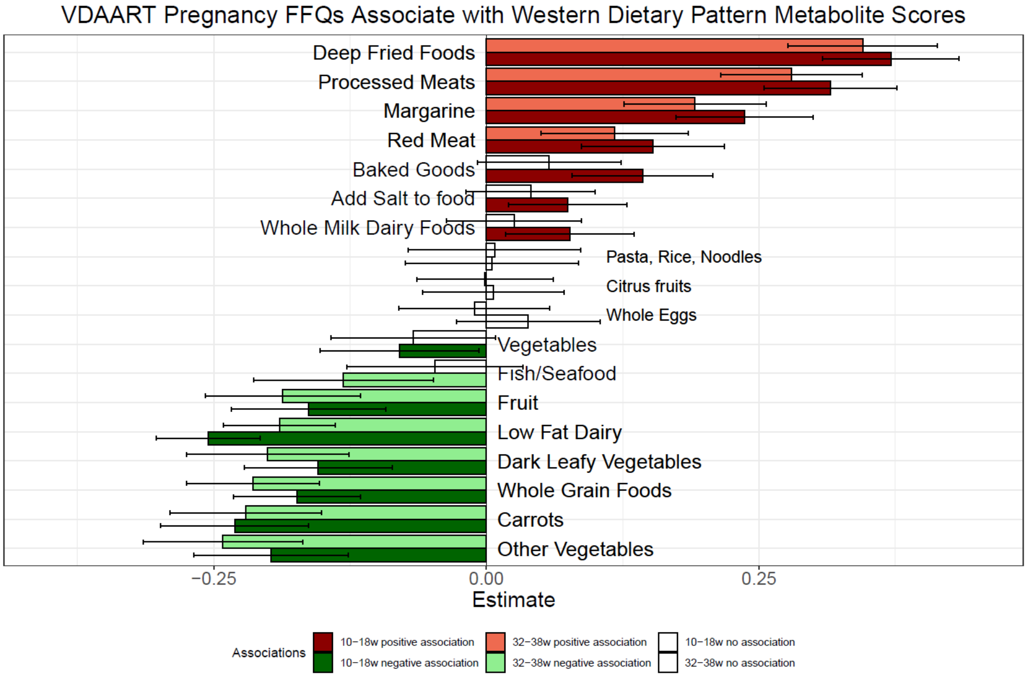

Line 215-221: Was any postnatal exposure controlled for as covariates such as socioeconomic status of the family during childhood, children's medical history, early education program attendance, etc.

Authors Response #9

Thank you for your comment regarding postnatal covariate selection. Our study focuses on the associations of early-life exposures, including maternal dietary patterns during pregnancy and breastfeeding duration, and thus we do not adjust for the postnatal exposures which you list above. Our rationale for this is that we carefully selected covariates based on causal reasoning, guided by a directed acyclic graph (DAG), to minimise confounding without inadvertently adjusting for colliders or introducing bias.

The suggested variables—such as socioeconomic status during childhood, children's medical history, and early education program attendance—are certainly relevant in broader contexts of child development research. However, in our study design, these variables may not be appropriate adjustments for the following reasons:

Socioeconomic Status During Childhood: Socioeconomic status (SES) during childhood may be influenced by early-life factors (e.g., maternal SES during pregnancy or parental income), which are already accounted for in our models. Adjusting for childhood SES could inadvertently adjust for a downstream effect of our primary exposures, potentially introducing collider bias. Children's Medical History: While children's medical history could influence cognitive outcomes, it may also be influenced by early-life exposures such as breastfeeding or maternal diet. Including this as a covariate could obscure the pathways we aim to study by adjusting for intermediate variables. Early Education Program Attendance: Similar to childhood SES, attendance in early education programs is likely influenced by parental decisions, which are, in turn, shaped by maternal SES and other factors during pregnancy. Adjusting for this variable risks introducing collider bias or over-adjustment.

Instead, our analytical framework prioritised the selection of covariates that would not disrupt the causal pathway between early-life exposures and long-term cognitive or growth outcomes, guided by a directed acyclic graph and supported by existing literature. By using this approach, we aim to provide an unbiased assessment of the associations under investigation while preserving the interpretability of our findings. We hope this response clarifies our rationale for covariate selection and addresses your concern.

The study also mentioned attendance of two RCTs. What was the proportion of participants who participated in RCTs? Can the excess exposure to nutrients included in the RCTs be adjusted for?

Authors Response #10

The COPSAC2010 cohort had two RCTs that commenced at the time of recruitment at 24 weeks of pregnancy.

Our Table 1, which details information of the 684 mothers that had available blood metabolome data available, shows that only 1 (0.1%) did not participate in the n3-LCPUFA trial and 108 (15.8%) did not participate in the Vitamin D trial.

The RCT interventions commenced after the clinical visit that the pregnancy food frequency questionnaires were filled in, and blood metabolome samples taken, and thus the RCT nutrient supplements taken throughout the 3rd trimester (high dose n3-LCPUFA and Vitamin D) would not directly affect our exposure variables.

To ensure robustness, we conducted sensitivity analyses adjusting for the effects of RCT participation (Supplementary Table 4). This approach allowed us to account for any residual influence of these interventions on the outcomes while preserving the integrity of our primary exposure assessments. These extra adjustments did not change the significance of our findings.

Thank you again for taking the time to review our work, we hope our responses have provided clarity and the revisions we have made have alleviated any residual concerns you may have.

Reviewer #3:

Thank you for the opportunity for reviewing this interesting manuscript. The study is well designed, and the paper is well-written. I have a few comments for the introduction and methods sections for the authors to improve the clarification of this manuscript.
Introduction:

* The authors mentioned "… but previous research has not adequately disentangled the potential independent, additive or pleiotropic effects of maternal and child dietary intake on brain morphological or cognitive outcomes." It would be helpful to list out what's known from the literature in a little bit more detail, so the readers could understand the current research gap.

* There is no mention of genetics in the intro. You may consider provide a brief summary of the literature on the genetic influence.

Authors Response #11

Thank you for reviewing our manuscript, and providing your comments which we hope to address adequately below.

We agree that providing additional context regarding the research gap in the Introduction is critical to help readers understand the rationale and significance of our study. Based on your suggestion, we have revised the Introduction to include a more detailed summary of previous studies and current knowledge gaps, as well as to incorporate a brief discussion of the meaningful genetic influences on neurodevelopmental outcomes.

Line 155: "*Given this critical period of rapid brain expansion during pregnancy (17,18), the interplay between nutrition, genetics and environmental factors is essential for supporting healthy neurodevelopment (19). Although genetics play a significant role in shaping neurodevelopmental outcomes, including cognitive abilities and head circumference (20,21), early-life environmental influences, such as maternal and child nutrition, may be influential during early development. Maternal diet, as assessed through isolated nutrients or food groups, has thus been shown to have long-term effects on cognitive development, intelligence, and behavioural outcomes (22,23). Focusing on overall dietary patterns rather than single nutrients, studies have found that children of mothers following unhealthy dietary clusters during pregnancy have lower IQ at age 8 compared to those born to mothers adhering to healthier, fruit- and vegetable-rich diets (24). Nutrition in early life, including breastfeeding, is another contributing factor to child neurodevelopment (25,26). However, previous research has not adequately disentangled the potential independent, additive or pleiotropic effects of maternal and child dietary intake on brain morphological or cognitive outcomes. Furthermore, whilst assessing individual nutrients or food groups is informative (27), this approach overlooks the potential synergistic effects of nutrients (28). Dietary patterns, however, offer a broader perspective on nutrient intake, capturing overall dietary habits and their potential interactions. Yet, reliance on food frequency questionnaires (FFQs) introduces recall bias (29), and few studies have mitigated this limitation by incorporating objective biomarkers (30).*"

This addition acknowledges the significant genetic contributions to neurodevelopment while emphasising the importance of early-life environmental influences, particularly maternal and child nutrition. We believe this enhances the manuscript by providing readers with a more comprehensive understanding of the research landscape and the gaps addressed by our study.

Additionally, while the Introduction outlines these critical aspects and helps frame our research question, we delve deeper into the broader context, including any potential genetic and environmental interplay, in the Discussion section. For example, we elaborate on our findings' independence from genetic contributions and link our findings to the wider literature base.

We hope this revised structure addresses your concerns and provides the necessary clarity to readers.

Methods:

* Is the metabolite score validated? If so, against what? It would be helpful to provide some background as the metabolite score is not widely used or known.

* The dietary exposure section may require a bit more details/ clarification. How was the metabolite score integrated with the dietary data collected in the FFQ? The authors mentioned the scores were modeled on dietary patterns identified by the FFQ. Was the residuals used for analysis? Later in the paragraph, the authors included "the metabolite scores were internally z-scored", which seems to serve as an independent exposure without integrating with the FFQ data. In general, the authors are expected to clarify how the metabolite information, FFQ data, were used to define the dietary patterns, as It was a bit unclear to me.

Authors Response #12

Thank you for your query regarding the dietary metabolite scores utilised in our manuscript. As mentioned above in response to Reviewer #1 and Reviewer 2 (see Authors Response #2 and #7). We have updated the main Methods text of our manuscript, to better describe the methodologies utilised to generate these metabolite scores. Previously, these were found in the `Supplementary Methods`, but we agree that given these are key exposures in the manuscript they should be visible to all readers in the main text.

Line 188 "*Our main exposures included dietary pattern metabolite scores, assessed from global blood metabolomics profiles taken at 24 weeks gestation (HD4 platform, Metabolon, Inc. (NC, USA)), derived from sparse partial least squares (sPLS) modelling, and breastfeeding duration (length of breastfeeding in days). Dietary pattern metabolite scores were modelled on dietary patterns during pregnancy, as identified from a validated 360 item semi-quantitative food frequency questionnaire (FFQ) completed by mothers at recruitment (24 weeks gestation), with a one-month recall period (29,30). Principal component analysis (PCA) identified dietary patterns based on calculated estimates of energy, macronutrients, and micronutrients derived from pregnancy food frequency questionnaires. The first principal*

component reflected a "Varied dietary pattern" (44.3%), while the second reflected a "Western dietary pattern" (10.7%), these dietary patterns were used to derive the metabolite scores in the sPLS models. Breastfeeding duration, measured as the length of breastfeeding in days, was also considered as a dietary exposure. Further details regarding the blood metabolomics analysis, derivation of dietary pattern metabolite scores via sPLS modelling, and data normalisation procedures for breastfeeding duration can be found in the Supplementary Methods. … Metabolite scores and breastfeeding length were internally z-scored at each timepoint, thus reflecting a change of 1 standard deviation within the population."

Briefly, the dietary metabolite scores were derived using sparse partial least squares regression, a form of machine learning, which aligns untargeted plasma metabolomics data with dietary patterns identified from principal component analysis (PCA) of food frequency questionnaires (FFQs). This integrative approach combines biochemical and self-reported dietary data to quantitatively reflect adherence to "Western" and "Varied" dietary patterns. These dietary patterns were identified based on energy, macronutrient, and micronutrient estimates from the FFQs, with quantitative metabolite scores derived from the PCA-derived dietary patterns. Regarding z-scoring, our aim was to standardise the exposures to ensure comparability across exposures and units, facilitating interpretation of effect sizes as changes per standard deviation. This approach enhances the generalisability of our results by allowing our findings to be more easily contextualised within the broader population and across studies.

With regard to our validation attempts, as described in the Discussion section, in a recent publication (recently "accepted" by Nature Metabolism) we have validated the Western dietary pattern metabolite score in an independent American mother-child cohort against pregnancy FFQs, demonstrating its robustness as a marker of dietary exposure (see figure below, shared from this manuscript). The use of metabolomics to validate dietary patterns is increasingly recognised in the literature as a robust approach to mitigating recall bias inherent to FFQs. We hope this additional context and the revisions made to the manuscript address your concern and clarify the methodologies used for the dietary metabolite scores.

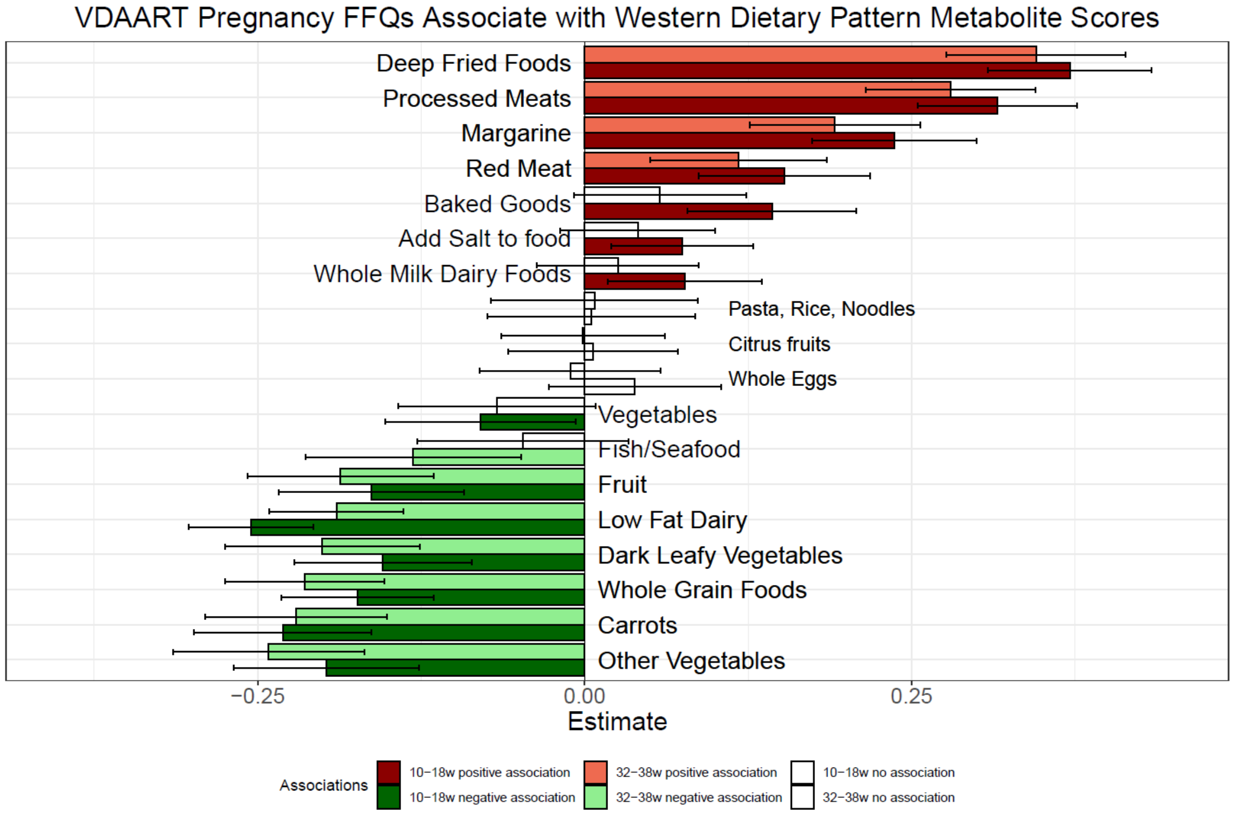

* The polygenic risk score section also need some more details. What loci were used to calculate the PRS? Was the method previously validated in your study population? How relevant was the PRS to your outcome of interest? Some of the information can be provided in the introduction, echoing a previous comment.

Authors Response #13

Thank you for your comment regarding the polygenic risk scores (PRS) methodology. We have revised the manuscript to address your concerns and provide additional details.

Line 245 ”*Maternal and child genotypes in the COPSAC2010 cohort were analysed using the Illumina Infinium HumanOmniExpressExome BeadChip. To calculate a polygenic risk score (PRS) for intelligence, a genome wide association study (GWAS) from the UK Biobank was utilised (n=391,124) measuring fluid intelligence (42). For child head circumference PRS scores, we utilised a GWAS derived from child head circumference measurements (n=29,192) (21). As COPSAC2010 participated in this consortium study, we contacted the authors who re-ran the GWAS with COPSAC2010 excluded. The PRS-CS method, which integrates millions of SNPs across the genome for enhanced precision, was applied. This method is well-validated in European populations and ensures that the PRS captures genome-wide genetic predispositions rather than being limited to specific loci. The strong genetic basis of intelligence and head circumference, combined with the robust GWAS methodologies, supports the relevance of these PRS to our outcomes. For head*

circumference, parameters were manually tuned during analysis using a grid search approach to optimise the global scaling factor. PRS were z-scored to mean 0 and SD 1 for interpretation."

Regarding validation, while the PRS-CS method has not been specifically validated in the COPSAC2010 cohort, it is a well-established and widely used method validated in European populations. Additionally, the original GWAS for intelligence and head circumference have large sample sizes and robust methodologies, ensuring the relevance of the PRS to our outcomes of interest. While validation of the PRS-CS method within the COPSAC2010 cohort was not explicitly considered in this study, high correlations between the polygenic risk scores and relevant outcomes can be observed in the heatmap presented in Supplementary Figure 2 (included below for convenience). This provides evidence of their relevance and applicability to the traits assessed in our analysis.

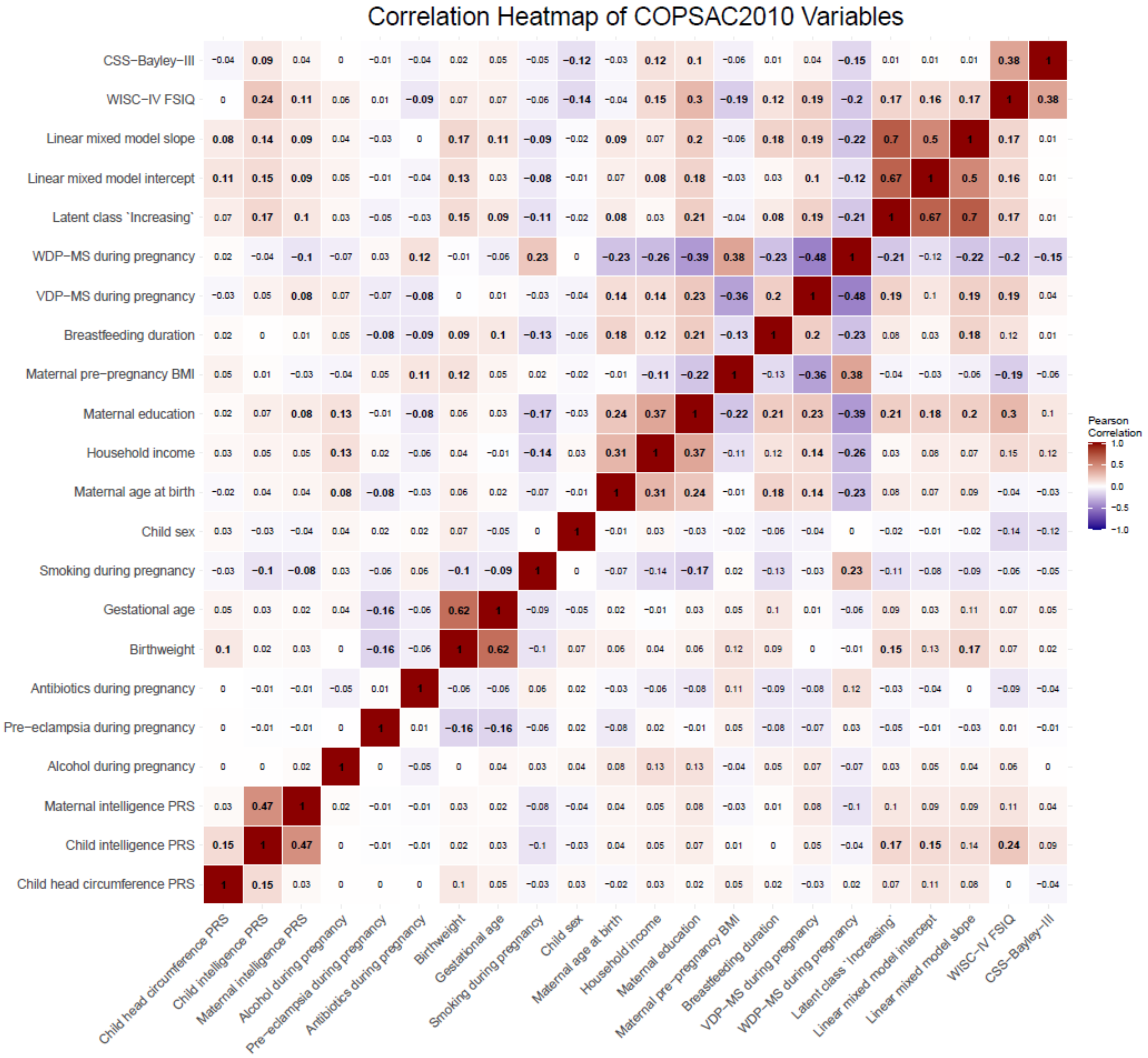

Covariates: why not considering parity as a covariate? It would be also important to clarify if the maternal dietary pattern was controlled for when assessing the relationships between child dietary score/ duration of breastfeeding and the outcomes, and vice versa.

Authors Response #14

Thank you for your insightful comments regarding covariate selection and clarification of maternal dietary pattern adjustments.

Regarding parity, our inclusion of covariates was guided by a directed acyclic graph (DAG) and supported by the existing literature. Based on this framework, parity was not included as a covariate because on our assessment of the literature we determined it to be neither a confounder nor directly influencing the causal pathway between pregnancy dietary exposures and child neurodevelopmental outcomes, such as cognition and head circumference growth. Parity may influence maternal or child characteristics, such as maternal age, but these are already accounted for in our models, making the inclusion of parity unnecessary and potentially leading to overadjustment without improving model validity.

To address your question regarding maternal dietary patterns, we have clarified this in the Dietary Exposures section of the Methods.

We have also included a statement regarding clarification of our modelling. Each exposure (Varied dietary pattern, Western dietary pattern and breastfeeding duration) was analysed independently with the stated model covariates, except in specific analyses where mutual adjustment was performed (see Line 373, Line 405, and Supplementary Figure 4). This approach allows for the examination of the direct effects of each exposure without introducing potential biases or confounding from overlapping exposures. We have updated our Methods text to reflect this clarification.

Line 205: "*When assessing the relationships between specific dietary exposures and neurodevelopmental outcomes, exposures were evaluated independently.*"

We believe these updates enhance the transparency of our methodological approach.

Thank you again for reviewing our manuscript.
